# Man versus machine? Self-reports versus algorithmic measurement of publications

**DOI:** 10.1371/journal.pone.0257309

**Published:** 2021-09-29

**Authors:** Xuan Jiang, Wan-Ying Chang, Bruce A. Weinberg

**Affiliations:** 1 Department of Economics, The Ohio State University, Columbus, OH, United States of America; 2 National Science Foundation, Arlington, VA, United States of America; 3 IZA, Bonn, Germany; 4 NBER, Cambridge, MA, United States of America; Iowa State University, UNITED STATES

## Abstract

This paper uses newly available data from Web of Science on publications matched to researchers in Survey of Doctorate Recipients to compare the quality of scientific publication data collected by surveys versus algorithmic approaches. We illustrate the different types of measurement errors in self-reported and machine-generated data by estimating how publication measures from the two approaches are related to career outcomes (e.g., salaries and faculty rankings). We find that the potential biases in the self-reports are smaller relative to the algorithmic data. Moreover, the errors in the two approaches are quite intuitive: the measurement errors in algorithmic data are mainly due to the accuracy of matching, which primarily depends on the frequency of names and the data that was available to make matches, while the noise in self reports increases over the career as researchers’ publication records become more complex, harder to recall, and less immediately relevant for career progress. At a methodological level, we show how the approaches can be evaluated using accepted statistical methods without gold standard data. We also provide guidance on how to use the new linked data.

## 1 Introduction

As the academic maxim goes, “Publish or perish.” In this environment, it is important to be able to measure the publication trajectories of scientists; how they vary across researchers, including by gender, race, ethnicity, immigrant status, and over a career; and how they relate to outcomes such as salary and promotions. Traditionally, to obtain productivity measures, researchers have turned to survey questions, but recently researchers have focused on using algorithmic approaches. However, little is known about the accuracy of these two sources of data.

Among the best survey data available for such analyses have come from the Survey of Doctorate Recipients (SDR). The National Center for Science and Engineering Statistics (NCSES) within the National Science Foundation has conducted the SDR biennially since 1973, collecting demographic information along with educational and occupational histories. Starting in 1995, self-reported data on publication counts for a reference period of five years have been collected. On the other side, an increasing number of algorithmically disambiguated databases have become available, from both academic and commercial sources. Examples include Dimensions, Elsevier, Microsoft Academic Graph, CiteSEER [1], Authority [2, 3]. One of the best-known commercial databases is the Web of Science.

In recent years, NCSES has enriched survey data with alternative data sources to increase the value of its data and to minimize the burden on survey respondents. One aspect of this work was an effort to link survey respondents from the 1993–2013 SDR to publications indexed by the Web of Science [4]. These linked data provide a unique opportunity to compare algorithmically disambiguated and matched data to survey data, including as determinants of research outcomes [5–9].

We use these newly available data on publications matched to the SDR to compare survey-based and algorithm-based measures of the publications of scientists. Our work makes four contributions. First, we study how publication counts compare across the two approaches both overall and for specific demographic groups, and we assess the relative quality of the two approaches. As we discuss later on, our results only apply to the WoS data, which has advantages and disadvantages relative to alternatives. Nevertheless, the WoS data are a prominent, respected dataset, and our analysis provides a valuable head-to-head comparison of one algorithmic approach to one survey approach to collect complex data that may be informative. Second, our analysis involves estimating how publication measures from the two approaches are related to career outcomes (e.g. salaries and faculty rank), estimates that are of interest in their own right. Third, as algorithmic approaches proliferate, so does the importance and cost of validation. The use of algorithmic data has increased rapidly in many research fields, for example, using names to impute gender, race, and ethnicity when demographic information is unavailable (e.g., [10]). While the conventional approach would be to collect a “gold standard,” something that is costly and extremely difficult to do well (e.g., even CV data contain mistakes), we lay out a radically different and far lower-cost approach to assessing data quality that others may find valuable. The utility of our approach has begun to be recognized, with [9] already applying it. As indicated [9], start from our basic approach and modify it by adding a manually-collected gold standard. Their basic approach answers a slightly different question from ours. Their work is best suited for assessing the signal-to-noise ratio in the WoS, which is more useful for assessing the quality of the WoS and working with the WoS data. Our approach compares the signal-to-noise ratio in the WoS to that in the SDR self-reports. Thus, it is better suited to assisting a researcher who is choosing between using one source of data versus the other. We also point to potential approaches using the data. At a methodological level, their approach requires the development of a gold standard, which is difficult to do accurately and costly (as a consequence, their main estimates are based on a sample that is roughly 7% the size of ours). Last, we provide guidance on how to use the new linked SDR-WoS data.

It is important to realize at the outset that the different approaches to obtaining publication data are likely to yield different information, results, and to generate some errors. First, there is likely to be pure measurement error in retrospective self-reports and these errors may be greater for people who are outside academia. We focus on academia in this paper in part because researchers in academia typically have more publications and therefore more data for matching. As a result, we have a larger dataset that links the self-reported publication and the algorithmic publication. Further, the career outcome measures, both salary and tenure status, are strongly predictive by publication for academia population; in contrast, the career outcomes for the non-academia population may be less associated with publication and more with other factors that are unobserved to researchers [11]. Second, self-reported publication counts, such as those included in the SDR, typically do not account for the quality of publications and are, therefore, a noisy measure of scientific contributions. Lastly, it is possible that there is what we might refer to as “bragging bias” in self-reports, with people who over-reporting their publications also over-reporting other outcomes such as salary (bragging may well be less common for other outcomes, such as tenure status, which is more discrete).

Another source of discrepancy between algorithmic data and self-reports is coverage: the target coverage of articles and journals in a database is defined, while self-reports cover all journal publications. (Of course, this defined coverage may or may not provide more uniformly quality.) Obviously, coverage also differs between self-reports and algorithmic data in that algorithmic data are, in principle, available to everyone, while self-reports are available only for those who respond to the survey, which excludes people who, during our analysis period, were outside of the United States and, obviously, anyone who had died.

The algorithmic approach also has limitations and errors, the most obvious of which are disambiguation and linking errors, which are likely to be greatest for relatively common names and when links must be made based on less information (e.g., without using e-mail addresses). Fortunately, for our comparison, disambiguation and linking errors as well as coverage issues stem from different sources than errors in self-reports. We have little reason to believe that the differences between the algorithmic data and self-reports will be highly correlated. Moreover, unlike the possible mis-reporting in the self-reports, the algorithmic measurement errors in the SDR-WoS dataset are unlikely to be correlated with over-reporting in outcomes. Our Methods section outlines the series of statistical approaches we used to assess the accuracy of the two data approaches.

Our findings are twofold. First, the overall results indicate that the potential biases in the SDR self-reports are likely smaller than those in the algorithmic data. While machine learning algorithms are improving rapidly over time (and the WoS is only one of many algorithmic approaches), this finding suggests that machine learning should not be viewed as categorically superior to self-reports. Second, while we find that overall the algorithmic approach underperforms self-reports, the errors in the two approaches are quite intuitive, which could be leveraged by researchers using the SDR data and guide users and producers of other data. Specifically, the accuracy of matching depends on the frequency of names and the data that were available to make matches (e.g., e-mail addresses). By subsetting the sample, we are able to show that the algorithmic data perform as well or better than the self-reports for people with uncommon names or for whom more data were available for matching. In contrast, the noise in self-reports is expected to increase over one’s career as a researcher’s publication records become more complex, harder to recall, and less immediately relevant for career progress (e.g., once researchers have permanent positions and have progressed up the academic career ladder).

Taken together, our findings suggest potential approaches for users of the SDR publication data. One natural approach is to run estimates using both sets of variables and triangulate the findings. Depending on the research question, a second option would be to subset the data to the groups for which a given measure is more precise. A third option is to instrument for one measure (e.g., the self-reported publications) using the other (e.g., the algorithmic measure). Obviously, the preferred approach will depend on the specific research question.

The algorithmic approach has at least three additional advantages. First, the direct linkage to publications makes it possible to include a range of measures of scientific impact (i.e., citations to articles), which would be hard to collect as part of a survey. These measures can be used to control for publication quality, although we have found that including WoS citation / impact measures does not have a meaningful effect on our salary or promotion estimates. Second, it is possible to use algorithms to obtain publications for researchers who are no longer living and for those for whom publications may be less salient (e.g. those in industry). Third, as indicated, using algorithmic data to attach publications reduces the burden on respondents, which might reduce the length of the survey or provide space for additional questions on other topics. On the other hand, it is important not to underestimate the cost of algorithmic linkages, which can be costly to conduct and, because the underlying data are proprietary, are not free to agencies.

## 2. Data

### 2.1 SDR and Thomson Reuters Web of Science™

The Survey of Doctorate Recipients (SDR) is a longitudinal survey of individuals who have earned a research doctorate in science, engineering, or health (SEH) field from a U.S. institution. Conducted by the National Center for Science and Engineering Statistics (NCSES), it contains a range of information on demographics, employment, and educational histories. The survey included questions about research outputs, including publications and patents, in 1995, 2003, and 2008.

To improve its understanding of the research output by U.S.-trained SEH doctorates, NSF collaborated with Clarivate Analytics to use machine learning to match SDR respondents to the authors of publications indexed by the WoS. Drawing directly on the description in [12], the matching algorithm incorporates name commonality, research field, education and employment affiliations, co-authorship networks, and self-citations to predict matches between SDR respondents and the WoS. Quoting directly from [5], the overall procedure consisted of five steps:

1. A gold standard data set was constructed for use in training prediction models and in validating predicted matches;
2. Candidate publications were identified and blocked using last name and first initial;
3. The Round One matching was conducted using Random Forests^TM^ (RF) [13] classification models trained to identify publications which could be matched to SDR respondents with a high degree of confidence. The high-confidence matches, called “seed publications,” were used to increase the amount of data available for the subsequent matching;
4. Data were extracted from the seed publications and combined with survey data used in Round One to enrich the RF models for increased recall and to make the final predictions;
5. The final matched data set was refined to ensure that no respondent was matched to more than one authorship on a single publication and that those with an exact match by email were considered matches.

### 2.2 Subset of SDR and WoS data used in this paper

To compare the quality of the SDR self-reported and WoS-linked publications, we analyze all three waves of SDR that included questions on publications and have been linked to WoS: the 1995, 2003, and 2008 waves. We present the results from the 2003 survey in the body of the paper. Results for the 1995 and 2008 waves are presented in the Appendix. The results are qualitatively similar across all three waves, with exceptions noted below.

**Fig 1:**
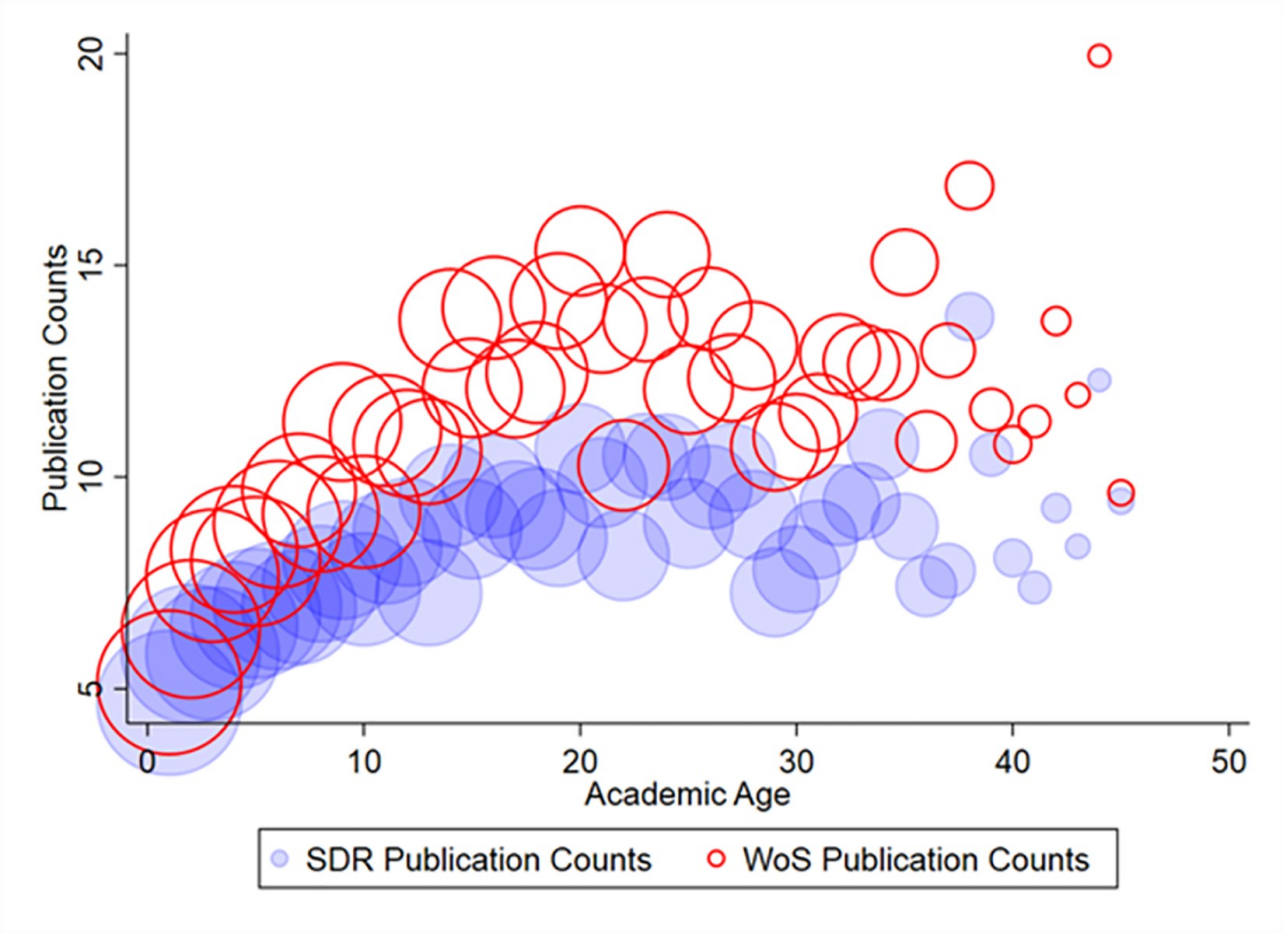
SDR and WoS publication counts by academic age. Notes: This figure shows the average publication counts by academic age from the two sources: the SDR and WoS from 1998–2003. The size of each bubble represents the size of the academic age group. Survey weights were used in creating this figure.

The 2003 SDR provides individual-level, self-reported data on journal article publication counts during 1998–2003. The SDR includes questions on both journal article publication counts and conference article publication counts. We only use journal article publication counts in this analysis. We compare these publication counts to the aggregated number of publications from the WoS in the same time frame. The SDR also provides comprehensive information on doctorate recipients, including demographic characteristics, academic records, and career outcomes. We focus on the sample of doctorate recipients working in academia. Table 1 shows summary statistics for the key variables used in this paper: publication counts from the SDR and WoS, self-reported salary, tenure status, and academic age in the sample. Tenure status is defined using self-reported faculty rank from the SDR. There are four faculty ranks: Assistant Professor, Associate Professor, Professor, and Other Faculty and Postdocs. We categorize Associate Professor and Professor as tenure = 1; and the rest as tenure = 0. The self-reported salary is in USD for the survey year (i.e., 2003 for the dataset we use in the main text). The lower panel of Table 1 compares SDR and WoS publication counts by sub-category, such as gender, race, and faculty rank. On average, WoS publication counts exceed SDR publication counts across the entire sample and all subgroups. The two outcome variables are the self-reported salary from the SDR and tenure status. The correlation coefficient between SDR publication counts and WoS counts is 0.68, indicating that they are somewhat but not highly correlated. Fig 1 plots SDR and WoS publication counts by academic age. Academic age is defined by number of years since the researcher received a Ph.D. It shows that WoS publication counts exceed those from the SDR at almost every academic age, except for the most senior ones.

**Table 1:** Summary statistics.

Variable	Obs	Weight	Mean	Std. Dev.
SDR Pub	10,738	238053.4	8.0	11.3
WoS Pub	10,738	238053.4	10.8	18.1
Salary	10,738	238053.4	74748.9	44173.6
Academic Age	10,716	237487.3	20.5	10.8
Tenure Status	10,738	238053.4	0.5446	0.498
**Male**				
SDR Pub	3,758	70938.5	6.3	8.4
WoS Pub	3,758	70938.5	8.1	15.0
**Female**				
SDR Pub	6,980	167114.9	8.8	12.2
WoS Pub	6,980	167114.9	11.9	19.2
**Race = Asian**				
SDR Pub	739	7487.5	7.9	11.1
WoS Pub	739	7487.5	8.5	13.8
**Race = Black**				
SDR Pub	637	7262.1	4.5	5.7
WoS Pub	637	7262.1	5.2	10.0
**Race = Hispanic**				
SDR Pub	1,344	29532.6	9.3	11.7
WoS Pub	1,344	29532.6	10.8	18.2
**Race = Others**				
SDR Pub	7,799	190300.7	8.0	11.3
WoS Pub	7,799	190300.7	11.1	18.4
**Race = White**				
SDR Pub	219	3470.5	9.2	14.2
WoS Pub	219	3470.5	11.2	21.5
**Faculty rank = “Other Faculty and Postdoc”**				
SDR Pub	2,379	51134.9	5.0	7.7
WoS Pub	2,379	51134.9	7.1	12.4
**Faculty rank = “Assistant Professor”**				
SDR Pub	2,352	46999.9	6.8	7.5
WoS Pub	2,352	46999.9	8.8	13.2
**Faculty rank = “Associate Professor”**				
SDR Pub	2,316	50983.0	7.5	9.3
WoS Pub	2,316	50983.0	9.7	13.8
**Faculty rank = “Professor”**				
SDR Pub	3,341	81509.4	11.5	15.0
WoS Pub	3,341	81509.4	15.5	24.5

Notes: This table shows summary statistics of the key variables used from the 2003 SDR and respondent’s SDR and WoS publication counts between 1998–2003.

**Fig 2:**
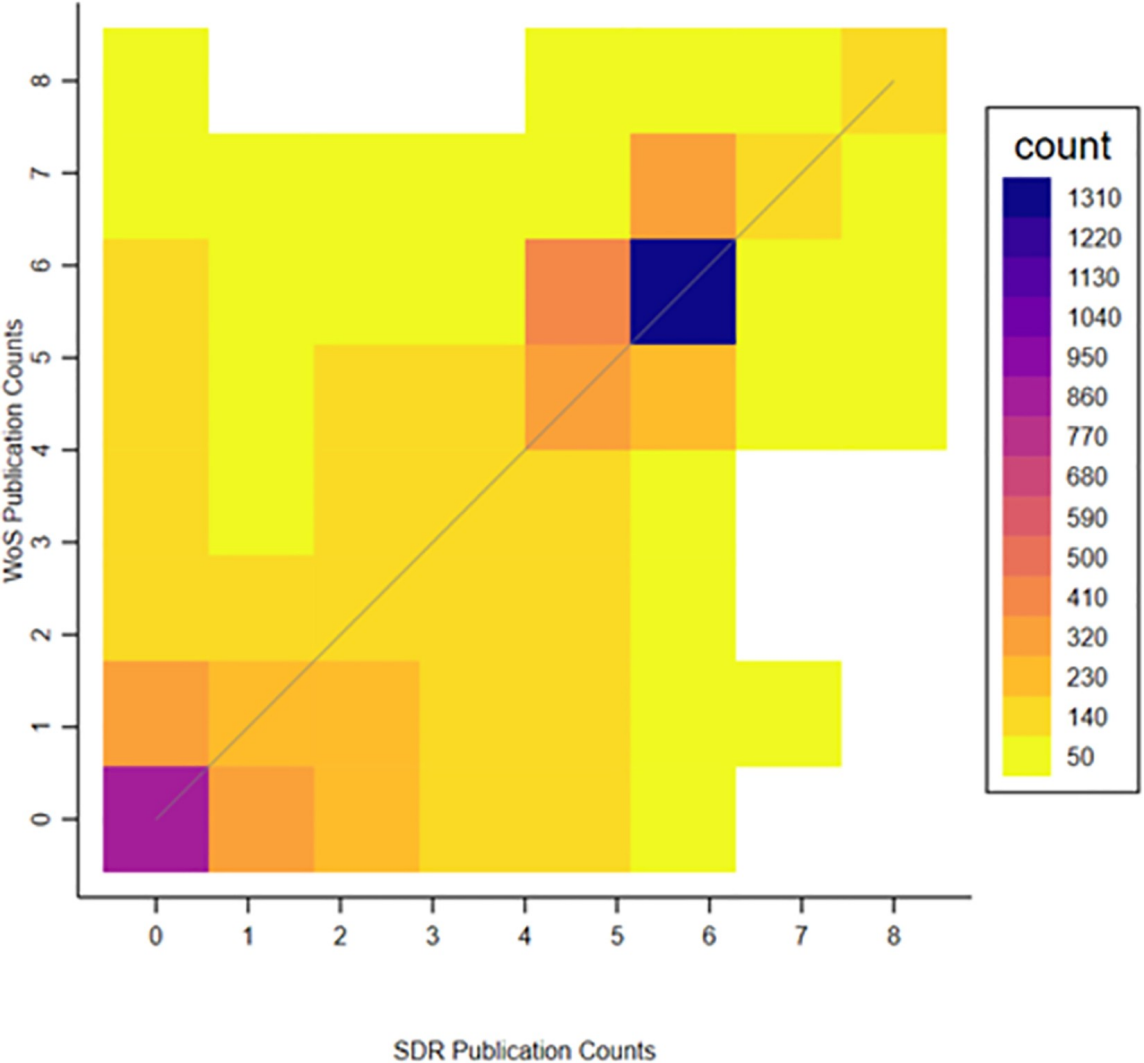
Relationship between the SDR and WoS publication counts. Notes: This figure shows the joint distribution of publication counts from the two sources: the SDR and WoS from 1998–2003. The colors represent the weighted sample size of each cell. Cells with fewer than 5 observations, including no observations, were excluded.

**Fig 3:**
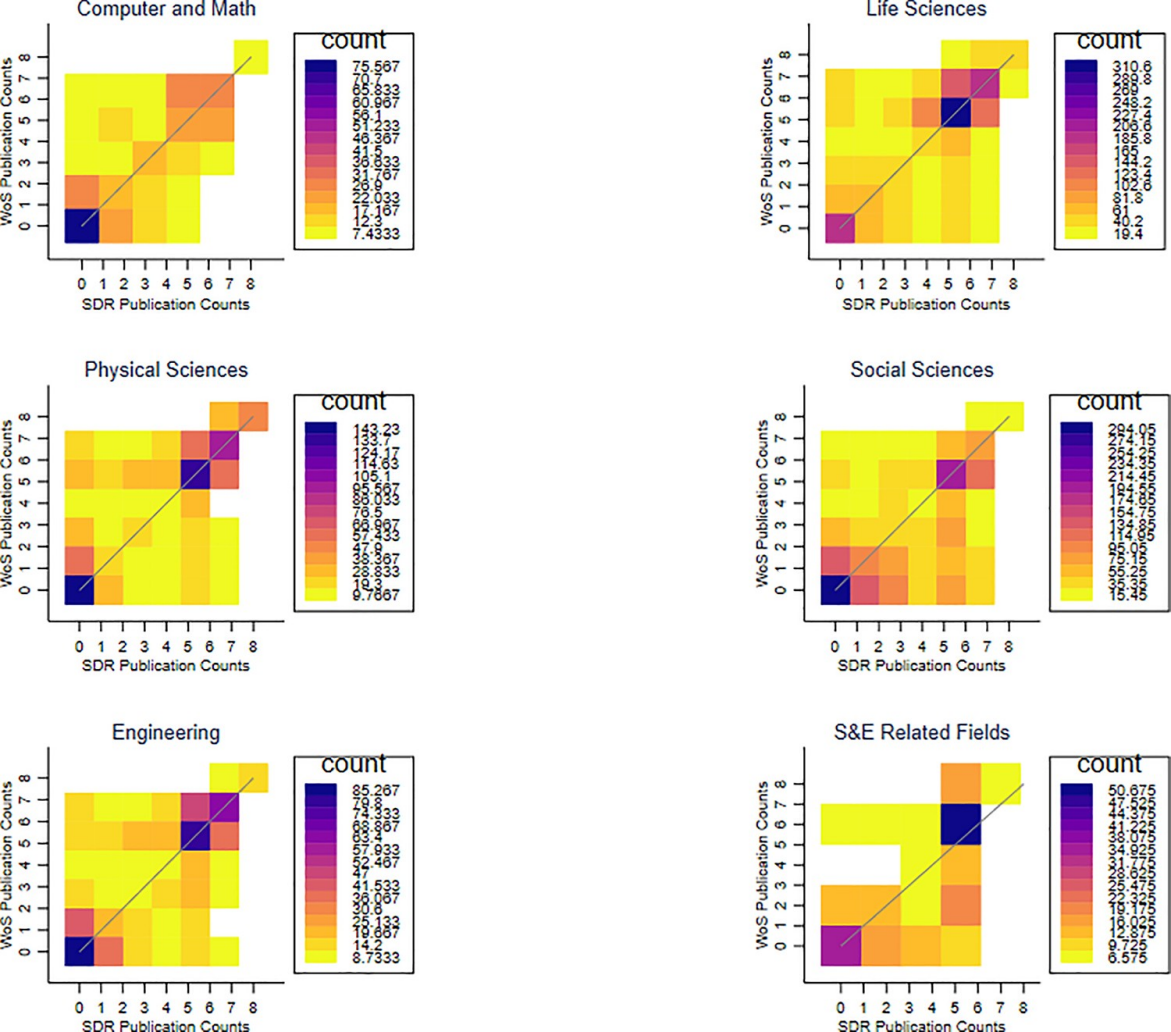
Relationship between the SDR and WoS publication counts, by research field. Notes: This figure shows the joint distribution of publication counts from the two sources: SDR and WoS from 1998–2003 by field. The colors represent the weighted sample size of each cell. Cells with fewer than 5 observations, including no observations, were excluded. There are 8 research field categories in the 2003 survey, and the 7^th^ (“Non-S&E Fields) and 8^th^ (Other Categories) were excluded due to small sample sizes.

To take a closer look at the relationship between the publication measures from the two sources, Fig 2 provides a heatmap of the joint distribution of SDR and WoS publication counts. The cells on the 45-degree line, where counts from both sources are the same, are most common, but there is also considerable dispersion. Fig 3 breaks down the joint distributions by research field. As in Fig 2, the most common pairings are along the 45-degree line, but there is considerable dispersion. Interestingly, the fields with the most researchers, including “Computer and Math”, “Life Sciences”, “Physical Sciences”, and “Engineering”, show larger WoS publication counts relative to SDR publication counts. By contrast, “Social Sciences” and “S&E Related Fields” tend to have larger SDR publication counts relative to WoS publication counts. This pattern also holds for the 2008 and the 1995 samples (see Figs A3 and A4 in S1 Appendix).

## 3 Methods

The heart of our work entails employing a standard set of econometric methods to address errors in the publication variables [14]. Our approach differs markedly from a more conventional comparison method, for instance, a manually constructed “gold standard,” and we believe it is more broadly applicable. Specifically, we analyze the relationship between a researcher’s self-reported career outcomes and the number of publications from both the SDR and WoS, to assess the quality of each measure of publication records. For our test case, the career outcomes, y_i_, for individual *i*, include self-reported salary and academic rank / tenure status. Outcomes are related to researchers’ own characteristics, X_i_, including demographics (e.g., gender, ethnicity, marital status, and age), experience, and field of study. This section lays out our conceptual framework and approach.

We start from the assumption that the true number of publications by researcher *i* is *P*_*i*_. Unfortunately, we do not observe the true number of publications. Rather, we observe the self-reported number of publications from the SDR, $P_{i}^{SDR}$, and the number of matched publications from the WoS, $P_{i}^{WoS}$. We can write $P_{i}^{SDR}$ and $P_{i}^{WoS}$ as functions of *P*_*i*_:

$$P_{i}^{SDR}=P_{i}+\varepsilon_{i}^{SDR}$$ (1A)

$$P_{i}^{WoS}=P_{i}+\varepsilon_{i}^{WoS}$$ (1B)

$\varepsilon_{i}^{SDR}$ and $\varepsilon_{i}^{WoS}$ are measurement errors in the number of SDR and WoS publications, respectively, which we regard as “errors-in-variables.” These are assumed to be uncorrelated with true publications, $Cov(P_{i},\varepsilon_{i}^{SDR})=Cov(P_{i},\varepsilon_{i}^{WoS})=0$. Because it is possible that the measurement error, especially in the SDR self-reports, is positively correlated with the true number of publications $\left(i.e.,Cov(P_{i},\varepsilon_{i}^{SDR})\neq 0\right)$, we discuss the sensitivity of our results to this violation of our assumptions.

The main source of error in the SDR is reporting error on the part of respondents, stemming from respondent recall. By contrast, the main sources of measurement error in the WoS data are algorithmic disambiguation and linking errors, which likely relate to the ambiguity of names and amount of information available to make matches. Thus, it seems plausible these two types of measurement errors are uncorrelated with each other: ${Cov(\varepsilon }_{i}^{SDR},\varepsilon_{i}^{WoS})=0$.

For much of what we do, we condition on a range of control variables. In this case, we augment Eqs (1A) and (1B) by including control variables represented by *X*_*i*_. In this formulation, we assume that $Cov(P_{i},\varepsilon_{i}^{SDR}|X_{i})=Cov(P_{i},\varepsilon_{i}^{WoS}|X_{i})=0$ and that ${Cov(\varepsilon }_{i}^{SDR},\varepsilon_{i}^{WoS}|X_{i})=0$.

### 3.1 Horse race test: Comparing coefficients

A “horse race” test is a simple, informal approach to compare the ability (i.e., explanatory power) of the two key measures of publication counts to explain the outcome variable (e.g., salary). Given that both measures are supposed to capture the same underlying true publication count, if one of the measures is a better predictor in the sense that it has a greater coefficient and stronger statistical significance, it indicates that it measures more precisely. [15, 16] employ similar logic, albeit in a very different context.

Specifically, consider a regression of some outcome *y*_*i*_, such as the natural logarithm of salary, on publications. With the true data on publications, that regression would be

$$y_{i}=\beta P_{i}+\gamma X_{i}+u_{i}$$ (2A)

We would interpret *β* as indicating how a greater number of publications is related to salary. (Although it is not critical for our analysis, we would be hesitant to give β a causal interpretation because salary and publications are both likely to be positively related to unobserved ability or motivation.)

Because we do not observe actual publications, we regress our outcomes, *y*_*i*_, on the SDR publication count or the WoS publication count, one at a time (i.e., separate regressions, (2B) and (2C)), and also include them both in the same regression (2D).


$$y_{i}=\beta^{SDR}P_{i}^{SDR}+\gamma X_{i}+u_{i}^{SDR}$$ (2B)

$$y_{i}=\beta^{WoS}P_{i}^{WoS}+\gamma X_{i}+u_{i}^{WoS}$$ (2C)

$$y_{i}=\beta^{SDR}P_{i}^{SDR}+\beta^{WoS}P_{i}^{WoS}+\gamma X_{i}+u_{i}^{Both}$$ (2D)

We compare the coefficients, *β*^*SDR*^ and *β*^*WoS*^. We note that the various *β*^*SDR*^ and *β*^*WoS*^ and *γ* estimates from (2B)–(2D) are analogous but different parameters.

Under the assumptions that measurement errors in our publication measures are uncorrelated with each other, ${Cov(\varepsilon }_{i}^{SDR},\varepsilon_{i}^{WoS}|X_{i})=0$, and with the error in (2A) $Cov(u_{i},\varepsilon_{i}^{SDR}|X_{i})=Cov(u_{i},\varepsilon_{i}^{WoS}|X_{i})=0$, standard errors-in-variables logic allows us to get a sense of the amount of measurement error in the two measures (in (1A) and (1B)) from the estimated coefficients *β*^*SDR*^ and *β*^*WoS*^ [14]. To lay out the effects of these errors-in-variables formally, consider one of the regressions (2B) or (2C) where we regress outcomes on $P_{i}^{j}$, where j∈{SDR, WoS}.


$$\hat{\beta^{j}}=\frac{Cov(y_{i},P_{i}^{j}|X_{i})}{Var(P_{i}^{j}|X_{i})}=\frac{Cov(y_{i},P_{i}+\varepsilon_{i}^{j}|X_{i})}{Var(P_{i}|X_{i})+Var(\varepsilon_{i}^{j}|X_{i})}=\beta \frac{Var(P_{i}|X_{i})}{Var(P_{i}|X_{i})+Var(\varepsilon_{i}^{j}|X_{i})}$$ (3)

Thus, relative to the true *β* in (2A), the error in the variable $P_{i}^{j}$ biases *β*^*j*^ toward zero with the downward bias depending directly on the amount of noise in the publication measure $P_{i}^{j}$ relative to the true variation in publications. We refer to this expression as the “errors-in-variables” formula. In the case where $Cov(P_{i},\varepsilon_{i}^{SDR})\neq 0,\;\hat{\beta^{SDR}}=\frac{\beta Var(P_{i}|X_{i})+Cov(P_{i},\varepsilon_{i}^{SDR}|X_{i})}{Var(P_{i}|X_{i})+Var(\varepsilon_{i}^{SDR}|X_{i})+2Cov(P_{i},\varepsilon_{i}^{SDR}|X_{i})}$, so the bias will not solely reflect the signal-to-noise ratio in $P_{i}^{SDR}$. (Naturally, an analogous relationship holds if $Cov(P_{i},\varepsilon_{i}^{WoS})\neq 0$.)

Returning to our maintained assumptions that $Cov(P_{i},\varepsilon_{i}^{SDR}|X_{i})=Cov(P_{i},\varepsilon_{i}^{WoS}|X_{i})=0$ and that the measurement errors in our publication measures are uncorrelated with each other ${Cov(\varepsilon }_{i}^{SDR},\varepsilon_{i}^{WoS})=0$, and with the error in (2A) $Cov(u_{i},\varepsilon_{i}^{SDR}|X_{i})=Cov(u_{i},\varepsilon_{i}^{WoS}|X_{i})=0$, we can compare, qualitatively, the amount of noise in each publication measure from the relative size of *β*^*SDR*^ and *β*^*WOS*^. Formally,

$$\frac{\hat{\beta^{SDR}}}{\hat{\beta^{WOS}}}=\frac{Var(P_{i}|X_{i})+Var(\varepsilon_{i}^{WoS}|X_{i})}{Var(P_{i}|X_{i})+Var(\varepsilon_{i}^{SDR}|X_{i})}$$ (4)

We can also assess the variance in publications. This can be done by noting that if ${Cov(\varepsilon }_{i}^{SDR},\varepsilon_{i}^{WoS}|X_{i})=0$, then

$${Cov(P}_{i}^{SDR},P_{i}^{WoS}|X_{i})={Cov(P_{i}+\varepsilon }_{i}^{SDR},{P_{i}+\varepsilon }_{i}^{WoS}|X_{i})=Var(P_{i}|X_{i})$$ (5)

Returning to the case where $Cov(P_{i},\varepsilon_{i}^{SDR}|X_{i})\neq 0$, Eqs (4) and (5) will not hold and ${Cov(P}_{i}^{SDR},P_{i}^{WoS}|X_{i})={Cov(P_{i}+\varepsilon }_{i}^{SDR},{P_{i}+\varepsilon }_{i}^{WoS}|X_{i})=Var(P_{i}|X_{i})+{Cov(P_{i},\varepsilon }_{i}^{SDR}|X_{i}),$ which would overstate the variance in *P*_*i*_.

We conduct three broad analyses–a baseline analysis, an analysis of different age groups, and an analysis based on the match quality in WoS–for which we have a series of indicators of match quality.

Underlying this analysis are four critical assumptions: that the measurement errors in our publication measures are uncorrelated with each other, ${Cov(\varepsilon }_{i}^{SDR},\varepsilon_{i}^{WoS}|X_{i})=0$, that each of the measurement errors is uncorrelated with the error in (2A), that $Cov(u_{i},\varepsilon_{i}^{SDR}|X_{i})=Cov(u_{i},\varepsilon_{i}^{WoS}|X_{i})=0$, and that $Cov(P_{i},\varepsilon_{i}^{SDR}|X_{i})=Cov(P_{i},\varepsilon_{i}^{WoS}|X_{i})=0$. As indicated, we believe that ${Cov(\varepsilon }_{i}^{SDR},\varepsilon_{i}^{WoS}|X_{i})=0$ is reasonable. We believe that assuming $Cov(u_{i},\varepsilon_{i}^{WoS}|X_{i})=0$ is also reasonable–a counter example would be that people with more ambiguous names might over- or under-report their salary. Given that East Asian names, especially Chinese and Korean names, are frequently among the most ambiguous, this cannot be excluded, but it also seems remote, especially because our baseline regressions control for race and ethnicity. If one of these assumptions were to be violated, the assumption $Cov(u_{i},\varepsilon_{i}^{SDR}|X_{i})=0$ seems like a likely candidate. We have referred to $Cov(u_{i},\varepsilon_{i}^{SDR}|X_{i})>0$ as “bragging bias,” meaning that people who over-report their salary may also over-report their publications. Such a violation would tend to bias $\hat{\beta^{SDR}}$ upward, which would make it appear to be less noisy than the errors-in-variables formula (given in Eq (3)) would suggest. Formally, in this case,

$$\hat{\beta^{SDR}}=\frac{Cov(y_{i},P_{i}^{SDR}|X_{i})}{Var(P_{i}^{SDR}|X_{i})}=\frac{Cov(y_{i},P_{i}+\varepsilon_{i}^{SDR}|X_{i})}{Var(P_{i}|X_{i})+Var(\varepsilon_{i}^{SDR}|X_{i})}=\frac{Cov(u_{i},\varepsilon_{i}^{SDR})+\beta Var(P_{i}|X_{i})}{Var(P_{i}|X_{i})+Var(\varepsilon_{i}^{SDR}|X_{i})}$$ (3′)

### 3.2 Instrumental variables analysis

Another approach is to employ instrumental variables. Instrumental variables analysis is a standard approach in many of the social sciences to address correlations between independent variables and the error term in a regression such as (2A)–(2D) [17–19]. There are two sources of bias in our estimates of *β*. One is the errors-in-variables in our $P_{i}^{j}$, for which instrumental variables analysis is ideal when there are two measures of a predictor variable that are subject to measurement errors, but with uncorrelated errors (i.e. ${Cov(\varepsilon }_{i}^{SDR},\varepsilon_{i}^{WoS}|X_{i})=0$). The other source of bias is the possible nonindependence of the measurement errors in salary and the measurement errors in publication, in which case the instrumental variable is also applicable when $\hat{\beta^{SDR}}$ is biased upward because of bragging bias, whereby people who over-report their salary also over-report their publications $Cov(u_{i},\varepsilon_{i}^{SDR}|X_{i})>0$. So long as $Cov(u_{i},\varepsilon_{i}^{WoS}|X_{i})=0$, instrumenting for $P_{i}^{SDR}$ with $P_{i}^{WoS}$ can also address this concern. At the same time, publications and salary both likely reflect “ability” and motivation, which are omitted from the models. Our instrumental variables strategy will not address this source of bias because ability is likely to be correlated with salary and both measures of publications.

Formally, we regress $P_{i}^{SDR}$ on $P_{i}^{WoS}$:

$$P_{i}^{SDR}=\delta^{WoS}P_{i}^{WoS}+\pi X_{i}+v_{i}^{SDR}$$ (6)

where the coefficient

$$\hat{\delta^{WoS}}=\frac{Cov(P_{i}^{SDR},P_{i}^{WoS}|X_{i})}{Var(P_{i}^{WoS}|X_{i})}=\frac{Cov(P_{i}+\varepsilon_{i}^{SDR},P_{i}+\varepsilon_{i}^{WoS}|X_{i})}{Var(P_{i}|X_{i})+Var(\varepsilon_{i}^{WoS}|X_{i})}=\frac{Var(P_{i}|X_{i})}{Var(P_{i}|X_{i})+Var(\varepsilon_{i}^{WoS}|X_{i})}$$ (7)

under the assumption that $\varepsilon_{i}^{SDR}\;and\;\varepsilon_{i}^{WoS}$ are uncorrelated. Thus, we can directly estimate the variance in the measurement error in the WoS publication measure relative to the variance in publications (i.e., the signal-to-noise ratio). The greater the measurement error, the greater the attenuation bias and the closer $\hat{\delta^{WoS}}$ will be to zero. In the case where $Cov(P_{i},\varepsilon_{i}^{SDR}|X_{i})\neq 0$, then $\hat{\delta^{WoS}}=\frac{Var(P_{i}|X_{i})+Cov(P_{i},\varepsilon_{i}^{SDR}|X_{i})}{Var(P_{i}|X_{i})+Var(\varepsilon_{i}^{WoS}|X_{i})}$ will overstate the signal to noise ratio in the SDR self-reports.

Of course, the same procedure can be run in reverse to obtain a measure of the signal to noise ratio in $P_{i}^{SDR}$, which generates a symmetric set of results. Specifically, if we estimate

$$P_{i}^{WoS}=\delta^{SDR}P_{i}^{SDR}+\pi X_{i}+v_{i}^{WoS}$$ (8)

we obtain

$$\hat{\delta^{SDR}}=\frac{Cov(P_{i}^{WoS},P_{i}^{SDR}|X_{i})}{Var(P_{i}^{SDR}|X_{i})}=\frac{Cov(P_{i}+\varepsilon_{i}^{W0S},P_{i}+\varepsilon_{i}^{SDR}|X_{i})}{Var(P_{i}|X_{i})+Var(\varepsilon_{i}^{SDR}|X_{i})}=\frac{Var(P_{i}|X_{i})}{Var(P_{i}|X_{i})+Var(\varepsilon_{i}^{SDR}|X_{i})}$$ (9)

This gives us an estimate of the measurement error in the SDR publication measure. In the case where $Cov(P_{i},\varepsilon_{i}^{SDR}|X_{i})\neq 0$, then $\hat{\delta^{SDR}}=\frac{Var(P_{i}|X_{i})+Cov(P_{i},\varepsilon_{i}^{SDR}|X_{i})}{Var(P_{i}|X_{i})+Var(\varepsilon_{i}^{SDR}|X_{i})+2Cov(P_{i},\varepsilon_{i}^{SDR}|X_{i})}$ will overstate the signal-to-noise ratio in the SDR self-reports.

Eqs (6) and (8) form the first stage of the instrumental variables estimation. The second stage of the instrumental variables procedure involves taking the prediction from the first stage and including it as an independent variable in a regression where the original outcome is the dependent variable. Specifically, we estimate models where outcomes are related to publications using both publication measures one by one as in Eq (1) and using instrumental variables to address measurement error. Formally, we first estimate Eq (6). We then regress the outcome variable, *y*_*i*_, on the fitted value from the first stage, $\hat{P_{i}^{SDR}}$

$$y_{i}={}^{SDR}\hat{P_{i}^{SDR}}+\gamma X_{i}+u_{i}\;(second\;stage)$$

The estimates from this model will tease out measurement error in $P_{i}^{SDR}$, even if the measurement in $P_{i}^{SDR}$ is correlated with the error in the salary equation, *u*_*i*_. We will have *β*^*SDR*^ = *β* so long as the error in the salary equation is not also correlated with the error in $P_{i}^{WoS},\;\varepsilon_{i}^{WoS}$. Intuitively, the instrumental variables procedure uses the portion of $P_{i}^{SDR}$ that is predicted by $P_{i}^{WoS}$, which is assumed to be uncorrelated with the error in the salary equation.

We can also implement this procedure in reverse by running Eq (8) as our first stage and then regressing the outcome on the predicted value from that equation in a second stage. This model will produce unbiased estimates under the assumptions that the measurement errors in the two publication measures are uncorrelated with each other and that the measurement error in $P_{i}^{SDR},\;\varepsilon_{i}^{SDR}$, is uncorrelated with the measurement error in the outcome equation, *u*_*i*_. Insofar as we may have some questions about the last assumption, this approach may be less compelling than the former (i.e., running Eq (6) as the first stage). At the same time, under the assumptions that ${Cov(\varepsilon }_{i}^{SDR},\varepsilon_{i}^{WoS}|X_{i})=0$ and that $Cov(u_{i},\varepsilon_{i}^{SDR}|X_{i})=Cov(u_{i},\varepsilon_{i}^{WoS}|X_{i})=0$, we have $\hat{\beta^{SDR}}=\hat{\beta^{WoS}}=\beta$ (that is, if both measurement errors are orthogonal to the error in the outcome equation, they will both be valid instruments and generate consistent estimates for *β*).

We note that if $Cov(P_{i},\varepsilon_{i}^{SDR}|X_{i})\neq 0$ then

$$\hat{\beta^{SDR}}=\frac{Cov(y_{i},P_{i}^{WoS}|X_{i})}{Cov(P_{i}^{SDR},P_{i}^{WoS}|X_{i})}=\frac{Cov(y_{i},P_{i}+\varepsilon_{i}^{WoS}|X_{i})}{Cov(P_{i}+\varepsilon_{i}^{SDR},P_{i}+\varepsilon_{i}^{WoS}|X_{i})}=\beta \frac{Cov(P_{i},P_{i}+\varepsilon_{i}^{WoS}|X_{i})}{Var(P_{i}|X_{i})+Cov(P_{i},\varepsilon_{i}^{SDR}|X_{i})}=\beta \frac{Var(P_{i}|X_{i})}{Var(P_{i}|X_{i})+Cov(P_{i},\varepsilon_{i}^{SDR}|X_{i})}$$

Thus, if $Cov(P_{i},\varepsilon_{i}^{SDR}|X_{i})\neq 0,\;\hat{\beta^{SDR}}$ will be biased downward with the bias being greater the greater the covariance between *P*_*i*_ and $\varepsilon_{i}^{SDR}$. Similarly, if $Cov(P_{i},\varepsilon_{i}^{SDR}|X_{i})\neq 0$ then

$$\hat{\beta^{WoS}}=\frac{Cov(y_{i},P_{i}^{SDR}|X_{i})}{Cov(P_{i}^{WoS},P_{i}^{SDR}|X_{i})}=\frac{Cov(y_{i},P_{i}+\varepsilon_{i}^{SDR}|X_{i})}{Cov(P_{i}+\varepsilon_{i}^{WoS},P_{i}+\varepsilon_{i}^{SDR}|X_{i})}=\beta \frac{Cov(P_{i},P_{i}+\varepsilon_{i}^{SDR}|X_{i})}{Var(P_{i}|X_{i})+Cov(P_{i},\varepsilon_{i}^{SDR}|X_{i})}=\beta \frac{Var(P_{i}|X_{i})+Cov(P_{i},\varepsilon_{i}^{SDR}|X_{i})}{Var(P_{i}|X_{i})+Cov(P_{i},\varepsilon_{i}^{SDR}|X_{i})}=\beta$$

Thus, $\hat{\beta^{WoS}}$ is consistent even if $Cov(P_{i},\varepsilon_{i}^{SDR}|X_{i})\neq 0$. This result is obtained because the measurement error in the instrument biases the first stage and the reduced form estimates similarly, leaving the second stage estimates consistent.

## 4 Findings

### 4.1 Results of the horse race tests

#### 4.1.1 Baseline

We first assess the explanatory power of the two publication measures for our two outcome variables using data from the 2003 SDR. Specifically, we estimate Eq (1) using the full sample of those who worked in the academic sector in the 2003 SDR. We replicate all analyzers using the 2008 and 1995 SDR as a robustness check. We estimate this model with three functional forms for the independent variables of interest: 1. The SDR publication counts and the WoS publication counts; 2. ln(SDR + 1) and ln(WoS +1); 3. ln(SDR + 1), ln(WoS + 1), and indicators of zero publications; 4. ln(SDR) and ln(WoS), and indicators of zero publications. When we use logarithmic measures of publications, i.e. ln(SDR) and ln(WoS), we set the log count variables equal to zero when the publication count is zero and include indicators for zero publications. In this procedure, the indicator for having zero publications gives the difference in outcomes between people with zero publications and one publication.

Table 2 shows the main results of the baseline analysis with ln(salary) as the outcome variable. We explore several regression specifications (each column is a separate regression model). Model (1) only includes the key variable, SDR publication counts and WoS publication counts. Model (2) adds in demographic controls including a female indicator, an indicator of marital status, linear and squared terms in academic age, and race indicators. Model (3) adds in field of study fixed effects using a coarse definition of field (7 fields in total). Model (4) switches to a fine definition of field (83 fields in total). Model (5) replaces the linear and squared terms in academic age with a full set of academic age fixed effects, to allow more flexibly for variation in experience. Model (5) is the richest model and is our preferred linear specification. Model (6) is the same specification as model (5) but replaces the SDR and WoS publication counts measured in levels with ln(SDR+1) and ln(WoS +1). Model (7) adds in indicators for zero SDR publications and zero WoS publications. Model (8) replicates model (7), replacing ln(SDR+1) and ln(WoS +1) with ln(SDR), ln(WoS).

**Table 2:** Horse race comparisons of SDR and WoS publication counts as determinants of salaries.

	(1)	(2)	(3)	(4)	(5)	(6)	(7)	(8)
SDR Pub	0.010***	0.008***	0.008***	0.008***	0.008***			
	(0.001)	(0.001)	(0.001)	(0.001)	(0.001)			
WoS Pub	0.004***	0.003***	0.004***	0.003***	0.003***			
	(0.001)	(0.000)	(0.001)	(0.001)	(0.001)			
ln(SDR Pub+1)						0.110***	0.102***	
						(0.012)	(0.011)	
ln(WoS Pub+1)						0.052***	0.066***	
						(0.007)	(0.008)	
ln(SDR Pub)								0.084***
								(0.010)
ln(WoS Pub)								0.055***
								(0.007)
Zero SDR Pub							-0.019	-0.073***
							(0.023)	(0.022)
Zero WoS Pub							0.053*	0.018
							(0.028)	(0.026)
Female		-0.084***	-0.081***	-0.074***	-0.073***	-0.059***	-0.059***	-0.060***
		(0.014)	(0.016)	(0.017)	(0.017)	(0.016)	(0.016)	(0.016)
Marital status		0.005	0.005	0.005	0.012	0.013	0.013	0.013
		(0.013)	(0.014)	(0.014)	(0.016)	(0.016)	(0.015)	(0.015)
Academic Age		0.042***	0.042***	0.042***				
		(0.003)	(0.003)	(0.003)				
Academic Age2		-0.001	-0.001	-0.001				
		(0.000)	(0.000)	(0.000)				
Asian		0.021	0.019	0.022	0.019	0.016	0.016	0.016
		(0.045)	(0.044)	(0.044)	(0.043)	(0.044)	(0.044)	(0.044)
Black		0.084	0.069	0.064	0.060	0.078	0.077	0.077
		(0.054)	(0.055)	(0.056)	(0.057)	(0.056)	(0.056)	(0.056)
Hispanic		0.053	0.046	0.039	0.040	0.034	0.034	0.034
		(0.045)	(0.044)	(0.042)	(0.042)	(0.042)	(0.043)	(0.043)
Others		0.037	0.037	0.039	0.039	0.032	0.032	0.032
		(0.042)	(0.041)	(0.041)	(0.042)	(0.042)	(0.042)	(0.042)
Field of Study FE (Coarse)	NO	NO	YES	NO	NO	NO		
Field of Study FE (Fine)	NO	NO	NO	YES	YES	YES		
Academic Age FE	NO	NO	NO	NO	YES	YES		
Observations	10716	10101	10101	10101	10101	10101	10101	10101
R-squared	0.049	0.132	0.142	0.158	0.164	0.176	0.176	0.176
Adjusted R-squared	0.049	0.131	0.141	0.150	0.153	0.165	0.165	0.164

Notes: The dependent variable in this table is the natural log of the self-reported salary in 2003. Each column is a separate regression, with different sets of control variables. Column (1) includes SDR and WoS publication counts in levels. Column (2) adds in demographic controls including an indicator of female, an indicator of marital status, linear and squared terms in academic age, and race indicators. Column (3) adds in field of study fixed effects using a coarse (7 field) definition. Column (4) replaces the coarse field of study fixed effects with a fine (83 field) definition. Column (5) replaces the linear and squared terms in academic age with a full set of academic age fixed effects. Column (5) is our preferred specification. Column (6) replicates column (5) but replaces publication counts from the SDR and WoS in levels with ln(SDR+1) and ln(WoS +1). Column (7) adds indicators for zero SDR and WoS publications. Column (8) replicates column (7), replacing ln(SDR+1) and ln(WoS +1) with ln(SDR) and ln(WoS), where we set the log count variables equal to zero when the publication count is zero. Standard errors are clustered at academic age level.

*p<0.1

**p<0.05

***p<0.01.

In general, we find that the 1998–2003 SDR’s publication count has substantially greater explanatory power for the 2003 base annual salary than the WoS publication count over the same time period. This relationship holds across all regression specifications: with or without demographic characteristics, regardless of the specification of academic age, controlling for coarse or fine research field classifications, and estimated in levels or either of the natural logarithm specifications for publication counts. Specifically, one SDR publication is associated with a 0.8% increase in salary, while one WoS publication is associated with a 0.3% increase in salary. The coefficient on SDR publications is more than double that on WoS publications.

Additionally, our estimates can provide some insights into how publications relate to outcomes along the intensive margin (i.e., how many publications a researcher has) and the extensive margin (i.e., whether a researcher has any publications). Specifically, column (7) shows that researchers with 0 WoS publications have earnings .053 log points *higher* than those with 1 WoS publication. Thus, there does not appear to be a penalty associated with having zero WoS publications. This finding may indicate that although the WoS failed to match any publications to such researchers, they actually have some publications, or that they are in fields or positions where WoS indexed publications are not particularly relevant. Given that researchers with zero WoS publications have higher earnings than those with 1 WoS publication, once we control for having zero WoS publications, the coefficient on ln(1+WoS publications) actually becomes slightly higher than in column (6). The estimates for zero SDR publications are negative, but imprecise, which makes it hard to assess whether there is a small penalty associated with having no SDR publications (i.e., the SDR extensive margin).

We replicate this table using the 1995 and 2008 rounds of the SDR, which also include self-reported publication counts, and find that the results are robust. See Table A2 in S1 Appendix. Interestingly, the magnitudes of the coefficients on the SDR publications are larger in the 2008 and 2003 rounds than in the 1995 round. By contrast, the magnitude of the coefficient on the WoS publications is quite similar across all three years. One potential explanation is that people are becoming better at keeping publication records and reducing measurement errors in self-reported publication count. Additionally, since Hirsch introduced the h-index in 2005 [20], there may be greater awareness of bibliometrics in the academic community. Another explanation is that the rate of return to publishing has increased, but that measurement error in the WoS has also increased, which we find less plausible because the quality of algorithmic disambiguation and linkage typically improve over time as more data elements become available.

Although salary is a natural measure of career outcomes, measurement error in the self-reported salary may be correlated with measurement error in the self-reported SDR publication counts. We then investigate an alternative measure of career outcomes, tenure status, which is presumably less subject to reporting error. We define tenure status using self-reported faculty rank (i.e., assistant professor, associate professor, professor, or other faculty and postdocs). While recognizing that policies differ somewhat across institutions, tenure status is defined as 1 if faculty rank is professor or associate professor and 0 otherwise. Although faculty rank may be misreported, because it is discrete and highly salient, it may be reported more accurately than salary, which is continuous and known to be reported with error. Table 3 reports estimates using tenure status as the dependent variable. The finding that SDR publication counts have more explanatory power than WoS counts is robust and even stronger than the results in Table 2.

**Table 3:** Horse race comparisons of SDR and WoS publication counts as determinants of tenure status.

	(1)	(2)	(3)	(4)	(5)	(6)	(7)	(8)
SDR Pub	0.007***	0.005***	0.005***	0.005***	0.005***			
	(0.001)	(0.001)	(0.001)	(0.001)	(0.001)			
WoS Pub	0.001**	0.000	0.000	0.0005*	0.0005*			
	(0.001)	(0.000)	(0.000)	(0.000)	(0.000)			
ln(SDR Pub+1)						0.063***	0.057***	
						(0.008)	(0.009)	
ln(WoS Pub+1)						0.008	0.007	
						(0.005)	(0.005)	
ln(SDR Pub)								0.045***
								(0.007)
ln(WoS Pub)								0.006
								(0.005)
Zero SDR Pub							-0.024	-0.058***
							(0.019)	(0.017)
Zero WoS Pub							-0.013	-0.015
							(0.018)	(0.017)
Female		-0.045***	-0.050***	-0.042***	-0.045***	-0.039***	-0.040***	-0.040***
		(0.011)	(0.011)	(0.010)	(0.010)	(0.009)	(0.009)	(0.009)
Marital status		0.016*	0.014*	0.014*	0.017**	0.017**	0.017**	0.017**
		(0.008)	(0.008)	(0.008)	(0.007)	(0.007)	(0.007)	(0.007)
Academic Age		0.071***	0.071***	0.070***				
		(0.003)	(0.003)	(0.003)				
Academic Age2		-0.001***	-0.001***	-0.001***				
		(0.000)	(0.000)	(0.000)				
Asian		0.054*	0.056**	0.060**	0.054**	0.054**	0.054**	0.054**
		(0.028)	(0.026)	(0.026)	(0.026)	(0.026)	(0.026)	(0.026)
Black		0.087***	0.074**	0.076***	0.076***	0.085***	0.086***	0.085***
		(0.032)	(0.029)	(0.028)	(0.028)	(0.028)	(0.028)	(0.028)
Hispanic		-0.002	0.006	0.016	0.012	0.010	0.010	0.011
		(0.025)	(0.024)	(0.024)	(0.024)	(0.024)	(0.023)	(0.024)
Others		0.024	0.027	0.028	0.025	0.024	0.024	0.024
		(0.024)	(0.023)	(0.023)	(0.023)	(0.023)	(0.023)	(0.023)
Field of Study FE (Coarse)	NO	NO	YES	NO	NO	NO		
Field of Study FE (Fine)	NO	NO	NO	YES	YES	YES		
Academic Age FE	NO	NO	NO	NO	YES	YES		
Observations	10366	9778	9778	9778	9778	9778	9778	9778
R-squared	0.035	0.426	0.444	0.462	0.476	0.483	0.483	0.482
Adjusted R-squared	0.035	0.426	0.443	0.457	0.469	0.475	0.476	0.475

Notes: The dependent variable in this table is an indicator of tenure status in 2003. Each column is a separate regression, with different sets of control variables. Column (1) includes SDR and WoS publication counts in levels. Column (2) adds in demographic controls including an indicator of female, an indicator of marital status, linear and squared terms in academic age, and race indicators. Column (3) adds in field of study fixed effects using a coarse (7 field) definition. Column (4) replaces the coarse field of study fixed effects with a fine (83 field) definition. Column (5) replaces the linear and squared terms in academic age with a full set of academic age fixed effects. Column (5) is our preferred specification. Column (6) replicates column (5) but replaces publication counts from the SDR and WoS in levels with ln(SDR+1) and ln(WoS +1). Column (7) adds indicators for zero SDR and WoS publications. Column (8) replicates column (7), replacing ln(SDR+1) and ln(WoS +1) with ln(SDR) and ln(WoS), where we set the log count variables equal to zero when the publication count is zero. Standard errors are clustered at academic age level.

*p<0.1

**p<0.05

***p<0.01.

#### 4.1.2 Academic age group heterogeneity

**Fig 4:**
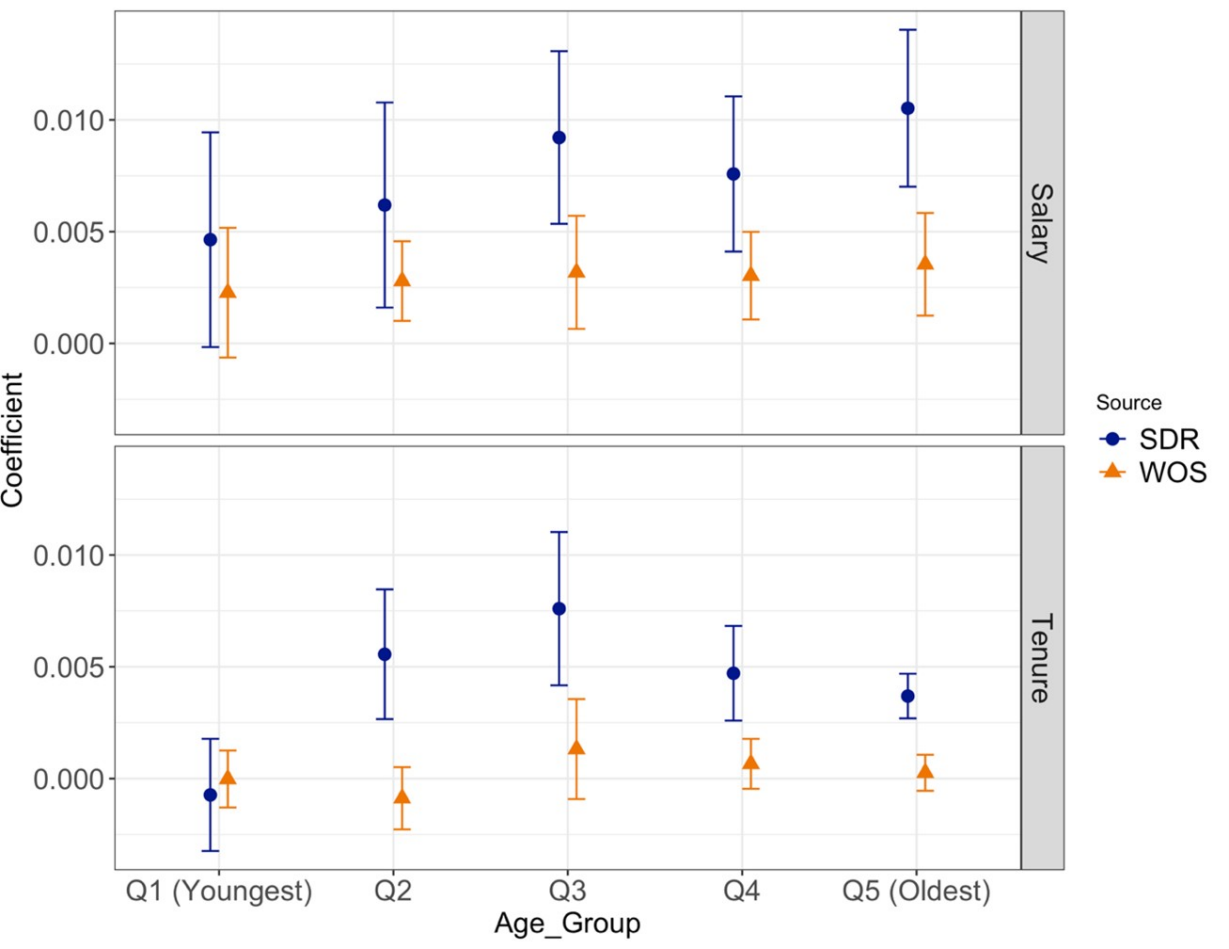
Relationship between outcomes and SDR and WoS publication counts by quintiles of academic age. Notes: This figure plots the coefficients and 95% confidence intervals of the coefficients from regressions of ln(Salary) (upper panel) and an indicator for tenure status (lower panel) on publication counts from the SDR and WoS using 2003 SDR data. Each panel reports estimates from 5 separate regressions stratified by academic age. From the left to right, the bins are [1,4], [5,9], [10,16], [17,25], [26,45].

We hypothesize that self-reported publication counts will become increasingly noisy over one’s career: as a researcher publishes more and moves into a senior author role, the precise number and timing of publications becomes harder to recall. If this is the case, self-reported publication counts are likely to degrade in quality later in a researcher’s career. By contrast, the accuracy of WoS’s algorithmically generated publication counts should not change over the career. These hypotheses lead us to expect that the coefficient on the SDR’s self-reported publication counts will decline over the career, while the coefficient on the WoS’s publication counts will be roughly stable, or even increase in specifications where the coefficients on the SDR publication counts decline. We run the horse race test by academic age quintiles. As shown in Fig 4, when we plot the coefficient on the SDR and WoS publication counts from five separate regressions, we find that the coefficient on the WoS’s publication count becomes more important as academic age increases. In particular, the coefficient on the WoS’s publication count is not statistically different from zero in quintile 1, when the dependent variable is ln(salary), shown in the upper panel, but is significantly different from zero in quintiles 2–5. Given that tenure status is a binary variable that has a specific relationship to academic age, the estimates for tenure status, shown in the lower panel, have something of a hump-shape, peaking at academic ages between 10 and 16, when many respondents are receiving tenure (e.g. after postdocs and/or second positions). At the same time, with tenure status as the outcome, the coefficients on WoS publications increase relative to those for SDR publications over the career.

#### 4.1.3 Match quality analysis

The quality of the WoS algorithmic publication counts likely vary according to the quality of WoS matches. Specifically, if WoS was unable to make a high-quality match, the WoS publication counts are likely to be noisy, and the coefficient on the WoS publication counts should be lower. By contrast, we do not expect the accuracy of the SDR’s self-reported publication counts to vary with WoS match quality, and therefore we expect the coefficients on the SDR’s self-reports to be essentially constant or perhaps increase slightly as WoS match quality deteriorates and hence the coefficient on WOS publication counts declines.

We explored two publication-level measures of the quality of WoS matches. First, the estimated probability that a publication matches the SDR respondent, as predicted by WoS’s random forest models, which is a continuous variable ranging between (0.5, 1). The WoS matches exclude articles with a predicted probability beneath .5. The higher the match probability, the more accurate the match on average. Second, the round in which the match was made, which indicates the number of rounds the random forest took to match the publication to the respondent. This is a discrete variable taking values of 0, 1 and 2, representing descending match quality: matched with an email address (value = 0), matched on round 1 (value = 1), and matched on round 2 (value = 2). The lower the number of match rounds, the higher quality the match. Both the match probability and match rounds are at the article level. To obtain an author-level measure, we take the mean of each variable across all of each respondent’s publications and use the author mean to proxy for WoS’s match quality. We also consider one person-level measure of match quality–the frequency that a particular respondent’s last name and first initial combination appears in the WoS database, which ranges from 1 to 218422. We expect that the lower the frequency, the higher the match quality. We estimate the horse race model across subsamples defined by quintile or quartile of each match quality proxy, including the average values across articles.

**Fig 5:**
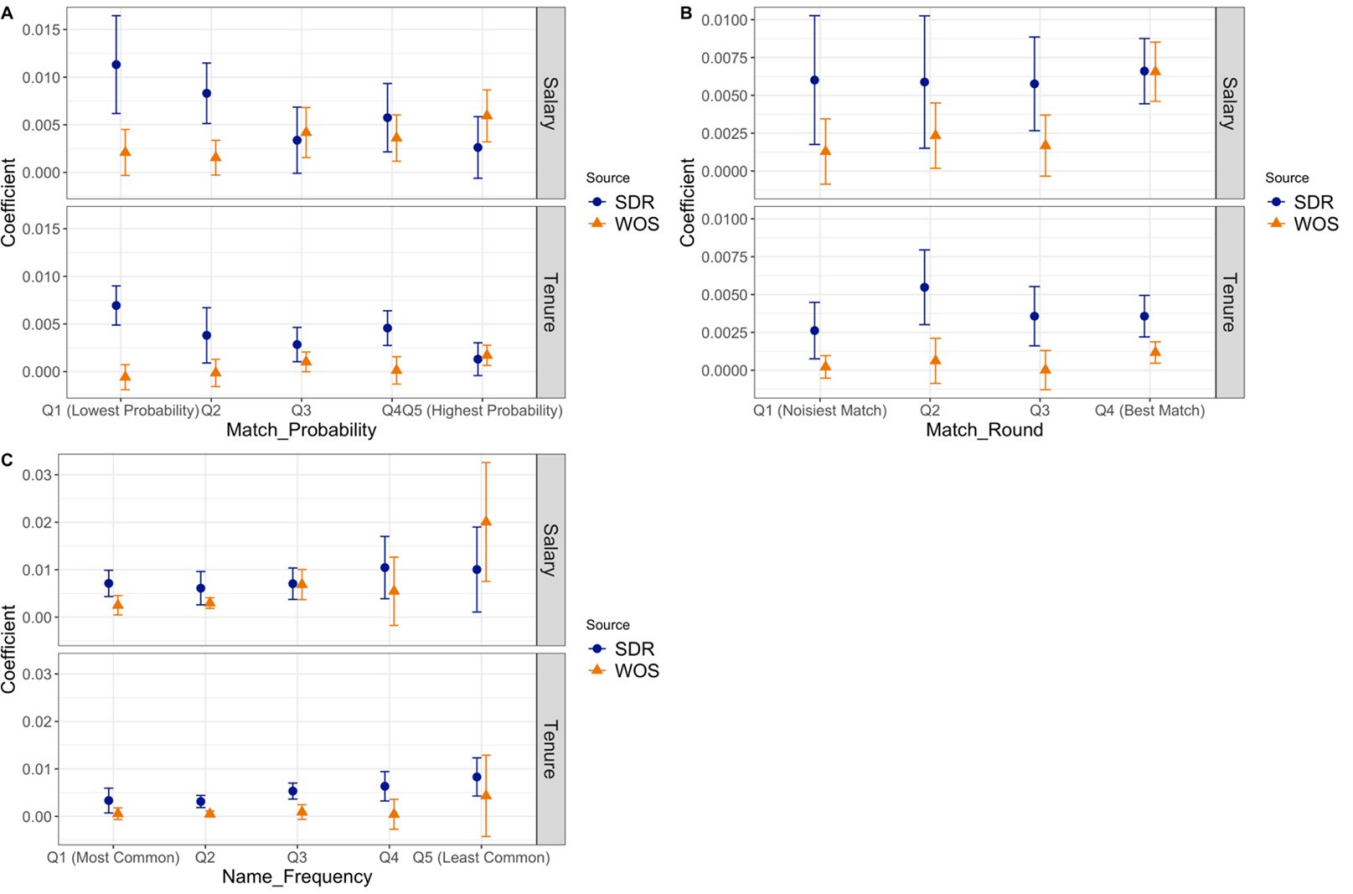
Relationship between outcomes and SDR and WoS publication counts by match quality measures. Notes: Each sub-figure plots the coefficients and 95% confidence intervals of the coefficients from regressions of ln(Salary) (upper panel) and an indicator for tenure status (lower panel) on publication counts from the SDR and WoS using 2003 SDR data. Each panel reports estimates from 4 or 5 separate regressions stratified by measures of match quality. In sub-figure A, estimates are stratified by the mean match probability among all of a respondent’s publications. From the left to right, the bins are [0.5000, 0.6901], [0.6902, 0.7568], [0.7568, 0.8128], [0.8128, 0.8693], [0.8693, 1]. In sub-figure B, the estimates are stratified by the mean match round of an author’s publications with lower match rounds representing higher confidence. From the left to the right, the bins are [1.768, 2], [1.3076, 1.767], [1, 1.3076], [0, 1]. (We stratify the data by quartiles instead of quintiles because the first quartile has a large cluster at 1.) In sub-figure C, the estimates are stratified by name frequency (first initial and last name of the SDR respondent), with higher frequency names being more ambiguous. From the left to the right, the bins are [1423, 218422], [282, 1423], [83, 281], [25, 82], [1, 24].

Fig 5, sub-figure A, shows different coefficients on the SDR’s publication counts and WoS’s publication counts across quintiles of match probability. Each sub-figure presents the regression estimates for each outcome variable: ln(salary) (upper panel) and tenure status (lower panel). We find that when the match probability is low (quintiles 1 or 2), WoS publication counts have significantly lower explanatory power than SDR publication counts. As the match quality gets higher, the coefficient on WoS publication counts increases and even exceeds the coefficient on SDR publication counts in some subgroups. This pattern is robust in the 2008 and 1995 datasets (see sub-figure A in Figs A9 and A10 in S1 Appendix).

A similar pattern emerges in Fig 5, sub-figure B, where the coefficient on the WoS publication counts is higher for people with more WoS publications that were matched in earlier rounds. Specifically, the coefficient on the WoS publication counts is not statistically different from the coefficient on SDR publication counts when the best matches could be made. The same pattern also shows in the results using the 2008 and 1995 data (sub-figure B in Figs A9 and A10 in S1 Appendix): the coefficient on the WoS publication counts is not statistically different from, or is even larger than, the coefficient on SDR publication counts when the best match or the second-best matches could be made. This finding reaffirms that the WoS publication count has larger explanatory power than that of SDR when WoS publications are matched with high quality.

Lastly, Fig 5, sub-figure C shows that for SDR respondents with common / ambiguous names, the coefficient on SDR publications is significantly larger than that on WoS publications, but when the frequency of a SDR respondent’s name is low (least common), the coefficient on WoS publication counts is generally not smaller than, and sometimes larger than, the coefficient on SDR publication counts. Again, Figs A9 and A10 in S1 Appendix show broadly similar results for the 1995 and 2008 SDR rounds. We have no explanation for the coefficient on WoS publications in the last column of sub-figure C of Figs A9 and A10 in S1 Appendix being negative.

Thus, across our three analyses, we find considerable and plausible differential explanatory power for WoS publication counts across different measures of WoS match quality. In general, the quality of the algorithmic data appears to be as good as the self-reported data when we restrict to the highest quality matches, although the overall quality of the algorithmic data is not as good as the self-reports. Still with improvements in matching algorithms, it seems plausible that algorithmic approaches have surpassed or will surpass many self-reports.

### 4.2 Results of the instrument variable analysis

We now compare the measurement errors in the two data sources using instrumental variables. We present the second stage and the first stage in the upper and lower panels of Table 4, respectively. We find both the deltas (coefficients in the first stage), *δ*^*SDR*^ and *δ*^*WoS*^, and the betas (coefficients in the second stage), *β*^*SDR*^ and *β*^*WoS*^, are statistically significant. Compared to the OLS estimates (in column (5) of Tables 2 or 3), the coefficients, *β*^*SDR*^ and *β*^*WoS*^, in the IV models (in the upper panel of Table 4) are significantly larger (more than double) the coefficients in the OLS analysis. This finding is important because it indicates that there is considerable measurement error in both *P*^*SDR*^ and *P*^*WoS*^.

**Table 4:** Instrumental variables analysis.

	(1)	(2)	(5)	(6)
**Second Stage**	**Salary**	**Salary**	**Tenure**	**Tenure**
SDR Pub	0.016***		0.006***	
	(0.020)		(0.001)	
WoS Pub		0.011***		0.005***
		(0.001)		(0.001)
N	10101	10101	9778	9778
R-sq	0.157	0.146	0.476	0.463
adj. R-sq	0.146	0.134	0.468	0.455
**First Stage**	**SDR Pub**	**WoS Pub**	**SDR Pub**	**WoS Pub**
SDR Pub		1.048		1.049
		(0.047)		(0.047)
WoS Pub	0.417		0.418	
	(0.019)		(0.020)	
**F-stat of the First Stage**	807.32	1762.56	863.08	1715.97

Notes: This table shows results from an instrument variables analysis. The upper panel reports second stage estimates and the lower panel reports first stage estimates. Each column is a separate model. In columns (1) and (2), the outcome variable is ln(Salary). In columns (3) and (4), the outcome variable is tenure status. The models in columns (1) and (3) use WoS publication counts to instrument for SDR publication counts. The models in columns (2) and (4) use SDR publication counts to instrument for WoS publication counts.

The magnitudes on the coefficients, *δ*^*SDR*^ *and δ*^*WoS*^, in the first stage are also informative. We find that *δ*^*WoS*^ is near 0.4 across all specifications, which is consistent with substantial measurement error. By contrast, *δ*^*SDR*^ is close to 1. Specifically, a low *δ* coefficient in the first stage (i.e. $\hat{\delta }<1$) is consistent with measurement error in publication counts. The lower estimate for *δ*^*WoS*^ compared to *δ*^*SDR*^ corroborates our results above, indicating that there is more measurement error in the WoS measures of publication counts than in the SDR measure.

One argument for using algorithmic data is that it is possible to obtain far more information on the quality of articles from algorithmic links than from self-reports. In other analyses, we have taken advantage of the measures of article quality (citation counts and the quality of journals) that are available in the WoS. Specifically, we add one of the following variables as a measure of article quality: the total number of citations in the past five years, the average number of citations per year, the total impact of the journal in the past five years, or the average impact of the journal in the past five years. These estimates do not show marked improvements in the explanatory power of the WoS data, which suggests that even the additional wealth of information in WoS does not offset its greater noise.

## 5. Conclusions

Machine learning provides a promising route to data linkage, which efficiently increases the utility of individual data sources and offers a wealth of research opportunities. This paper has explored a case of importance to the scientific community in which both algorithmic and survey responses are available and can be compared. Specifically, we focus on measures of scientists’ publications and how they relate to career outcomes measured by salary and tenure status / faculty rank. Perhaps surprisingly, we find that overall the publication counts generated by machine learning are more noisy than self-reports. Moreover, we find that the relative noise in the two sets of measures varies in an intuitive way. That is, self-reports degrade for senior researchers who have more publications and for whom it may be harder and less important to recall the exact quantity and timing of publications. Algorithmic measures degrade when names are more common or less data is available to make high-quality matches.

These findings are valuable for researchers using the publication variables in the SDR. A first approach is to run estimates using both sets of variables and triangulate the findings. Second, depending on the research question, one might subset the data to the groups for which a given measure is more precise. A third option is to instrument for one measure (e.g., the self-reported measure) using the other (e.g., the algorithmic measure). Obviously, the preferred approach will depend on the specific research question.

Other known databases, such as Dimensions, Microsoft Academic Graph, or Scopus, have different coverage of publications and/or authors and/or take a different disambiguation approach, and thus have different qualities ([21, 22], and references therein). In their abstract, [22] summarize their comparison of Dimensions, Scopus, and the Web of Science: “The results indicate that the databases have significantly different journal coverage, with the Web of Science being most selective and Dimensions being the most exhaustive.” Their cross-country analyses show that the focus on selectivity over breadth in the Web of Science is far more consequential for non-U.S. based researchers, who are out of our sample. Another approach would be to use researcher-curated databases such as Google Scholar or ORCiD. While even researcher-curated data are not perfect, there may be some advantages to these data for researchers who curate their profiles and keep them updated. On the other hand, our own analyses suggest that participation in these systems is far from complete. Ultimately, we analyze the WoS because it is the only database for which links to the SDR self-reported publications are available. While the use of one specific dataset is a limitation, the WoS is a prominent and widely-used dataset and its focus on selectivity over coverage is a smaller disadvantage (and perhaps even an advantage) for U.S.-based researchers. Despite these tradeoffs, we believe that our analysis contributes to the scarce literature on comparing self-reported and algorithmic data, and our methods could easily be applied to the other datasets if/when the NSF commissions such links.

Stepping back from our specific context, our results may be useful for data producers. They suggest that despite the understandable enthusiasm for them, even high-quality algorithmic approaches are not yet uniformly superior to self-reports. At the same time, we expect algorithmic approaches to improve over time relative to survey methods. Another promising approach that might be explored is recommendation systems, where algorithmic methods are used to populate lists for people to accept or reject. The ORCiD system (https://orcid.org/) is one prominent example of such an approach. Such an approach has the potential to reduce the burden on survey respondents at the same time that it allows for training data that can be used to further refine algorithmic approaches.

We believe that our underlying approach is broadly applicable. The conventional approach to assessing data quality is to manually build a “gold standard,” but this can be quite time-consuming and costly. Our approach permits estimation of data quality without requiring a gold standard using accepted statistical methods.

## Supporting information

S1 Appendix (DOCX)
