# Supplementary material for: Man versus machine? Self-reports versus algorithmic measurement of publications
Source: PLoS One. 2021 Sep 29;16(9):e0257309. doi: 10.1371/journal.pone.0257309 (PMC8480886; doi:10.1371/journal.pone.0257309)
Supplement: S1 Appendix — (DOCX) [file pone.0257309.s001.docx]

**Man Versus Machine? Self-Reports Versus Algorithmic Measurement of Publications**

**Appendix: Robustness Checks – Replications Using SDR 2008 and 1995**

Xuan Jiang*, The Ohio State University

Wan-Ying Chang, National Science Foundation

Bruce A. Weinberg, The Ohio State University, IZA, and NBER

*Corresponding author

Email: jiang.445@osu.edu


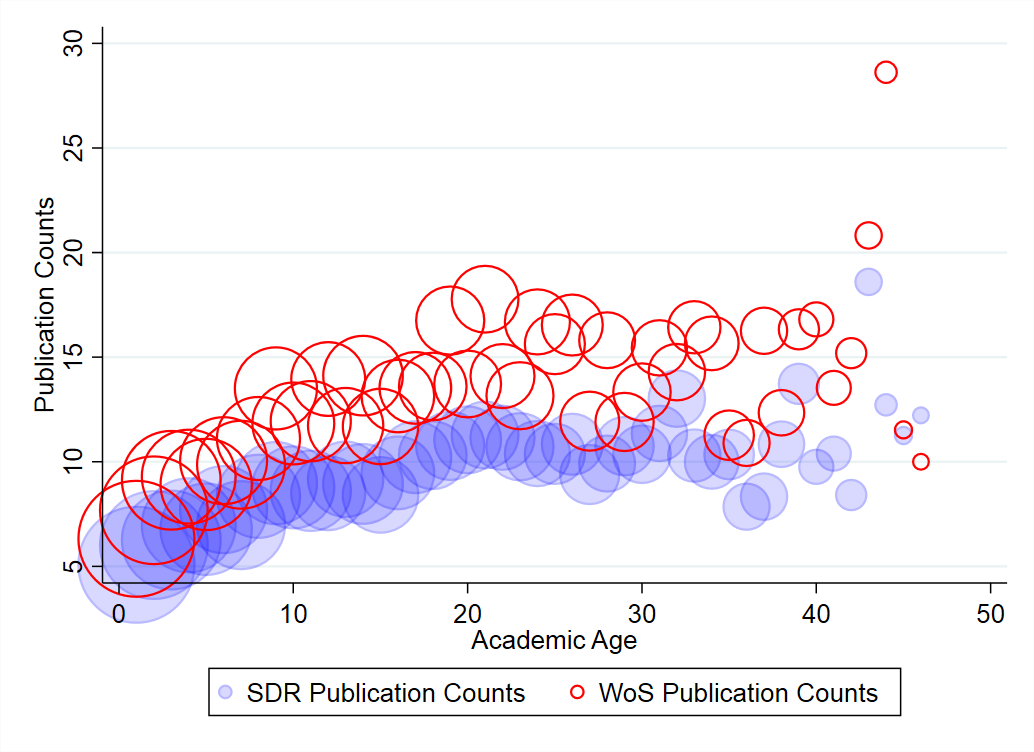


## Fig A1: SDR and WoS Publication Counts by Academic Age (2003—2008)

Notes: This figure shows the average publication counts by academic age from the two sources: the SDR and WoS from 2003-2008. The size of each bubble represents the size of the academic age group. Survey weights were used in creating this figure.


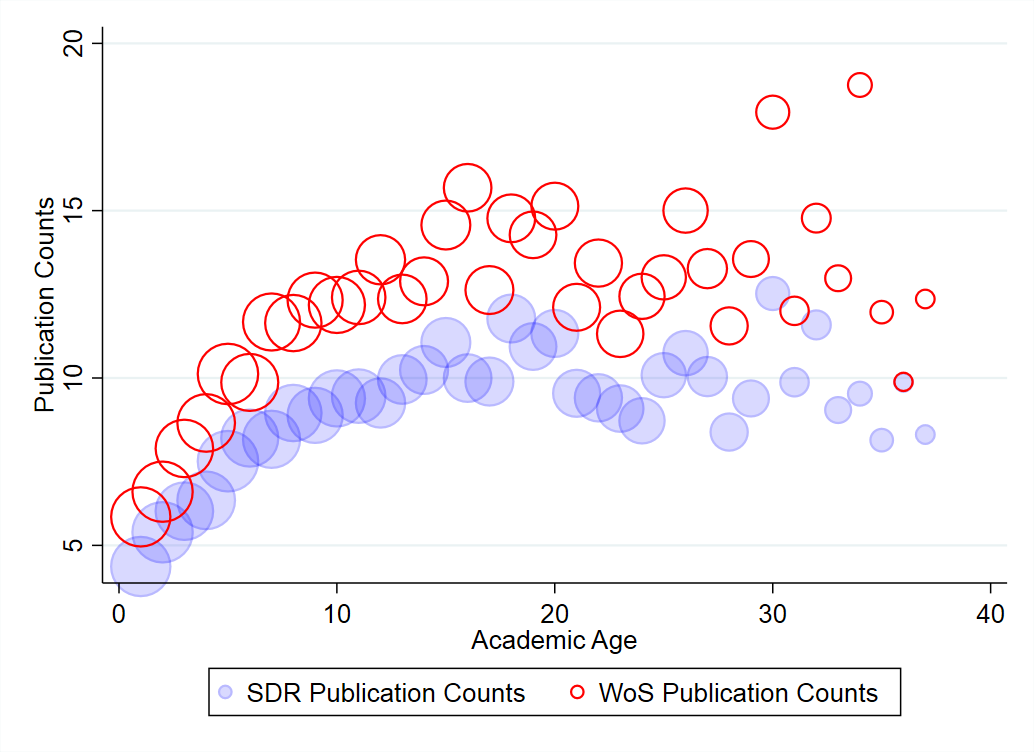


## Fig A2: SDR and WoS Publication Counts by Academic Age (1990—1995)

Notes: This figure shows the average publication counts by academic age from the two sources: the SDR and WoS from 1990--1995. The size of each bubble represents the size of the academic age group. Survey weights were used in creating this figure.


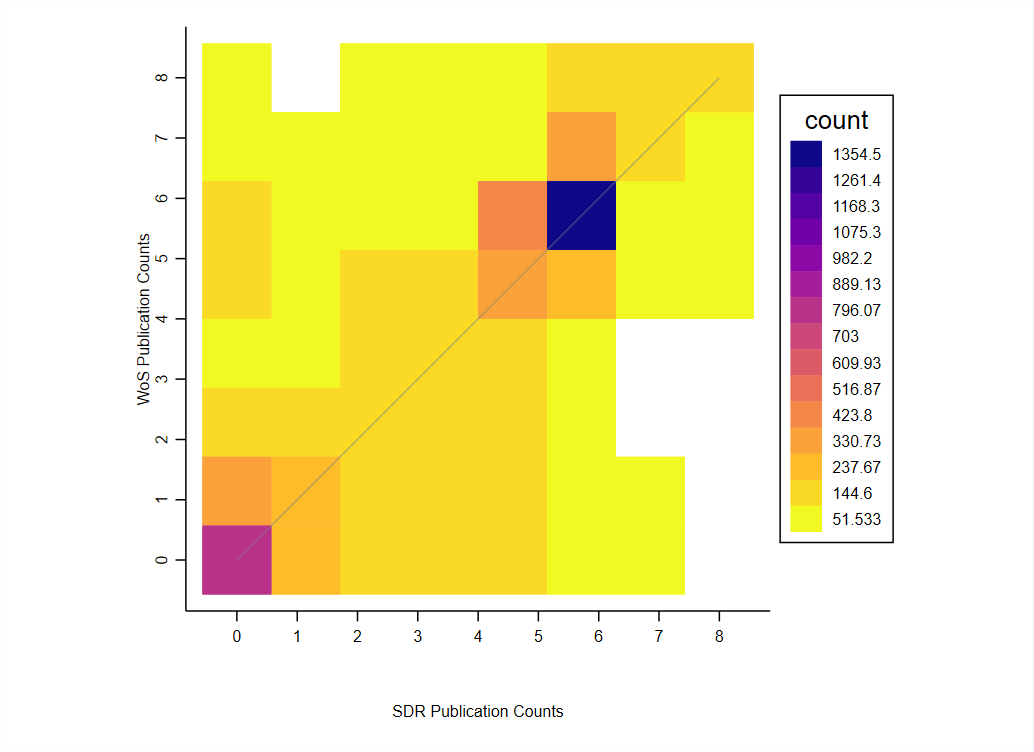


Fig A3: Relationship between the SDR and WoS Publication Counts (2003—2008)

Notes: This figure shows the joint distribution of publication counts from the two sources: the SDR and WoS from 2003—2008. The colors represent the weighted sample size of each cell. Cells with fewer than 5 observations, including no observations, were excluded.


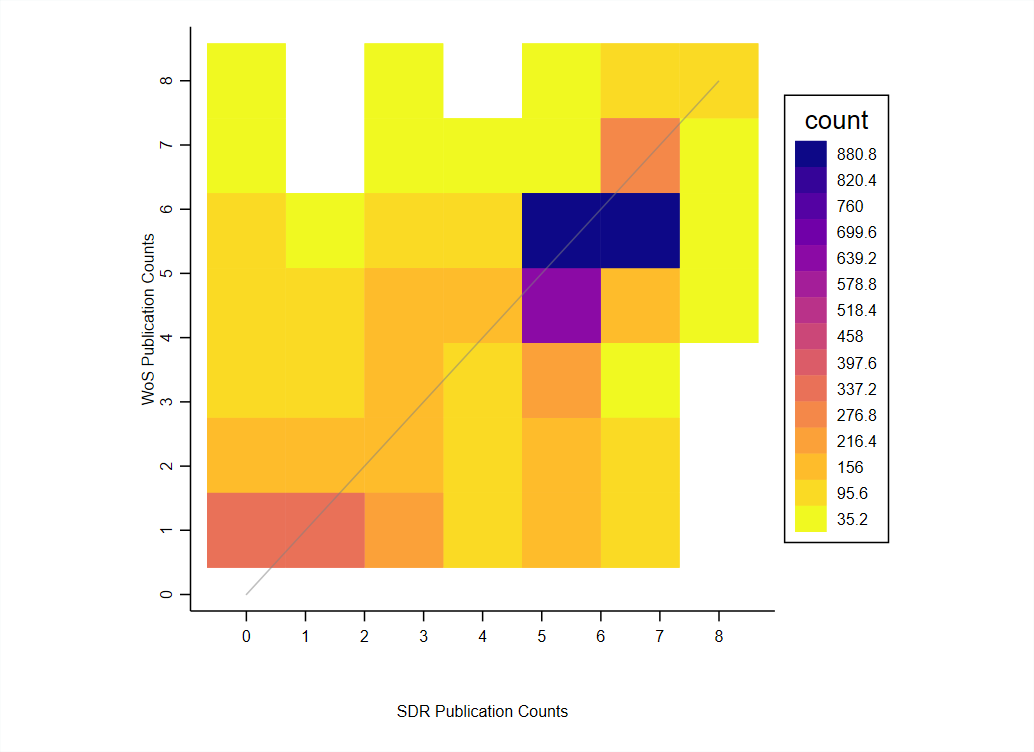


Fig A4: Relationship between the SDR and WoS Publication Counts (1990—1995)

Notes: This figure shows the joint distribution of publication counts from the two sources: the SDR and WoS from 1990—1995. The colors represent the weighted sample size of each cell. Cells with fewer than 5 observations, including no observations, were excluded.


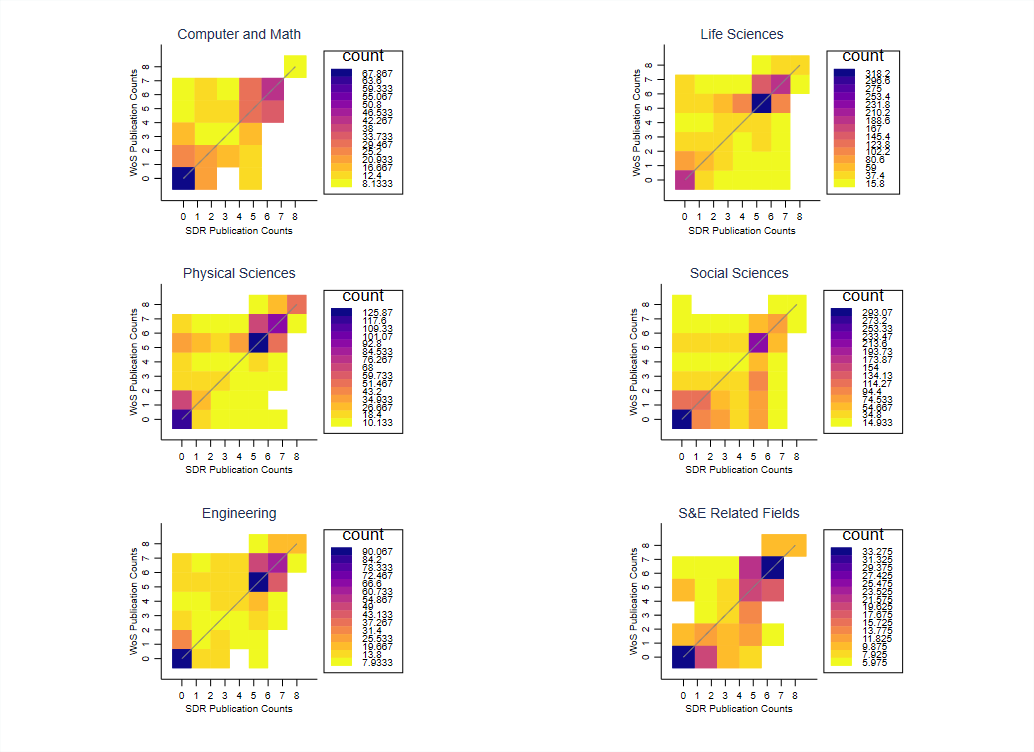


Fig A5: Relationship between the SDR and WoS Publication Counts, by Research Field (2003—2008)

Notes: This figure shows the joint distribution of publication counts from the two sources: SDR and WoS from 2003—2008 by field. The colors represent the weighted sample size of each cell. Cells with fewer than 5 observations, including no observations, were excluded. There are 8 research field categories in the 2008 survey, and the 7^th^ (“Non-S&E Fields) and 8^th^ (Other Categories) are excluded due to small sample size.


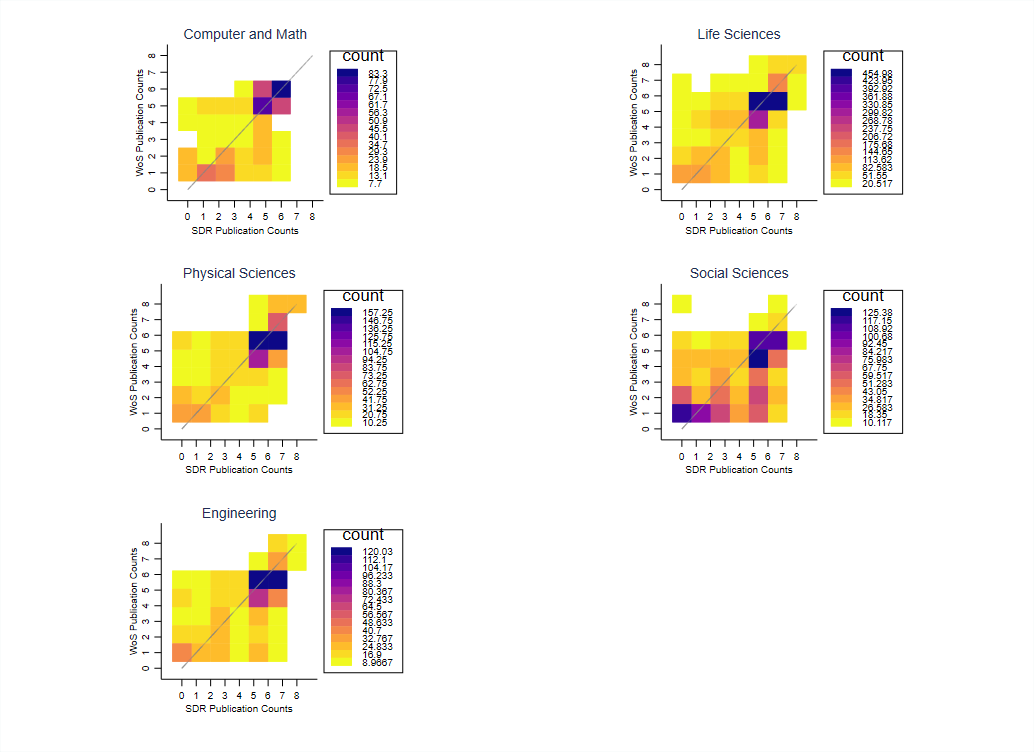


Fig A6: Relationship between the SDR and WoS Publication Counts, by Research Field (1990—1995)

Notes: This figure shows the joint distribution of publication counts from the two sources: SDR and WoS from 1990—1995 by field. The colors represent the weighted sample size of each cell. Cells with fewer than 5 observations, including no observations, were excluded. There are 6 research field categories in the 1995 survey, and the 6^th^ (“Non-S&E Fields) was excluded due to small sample size.


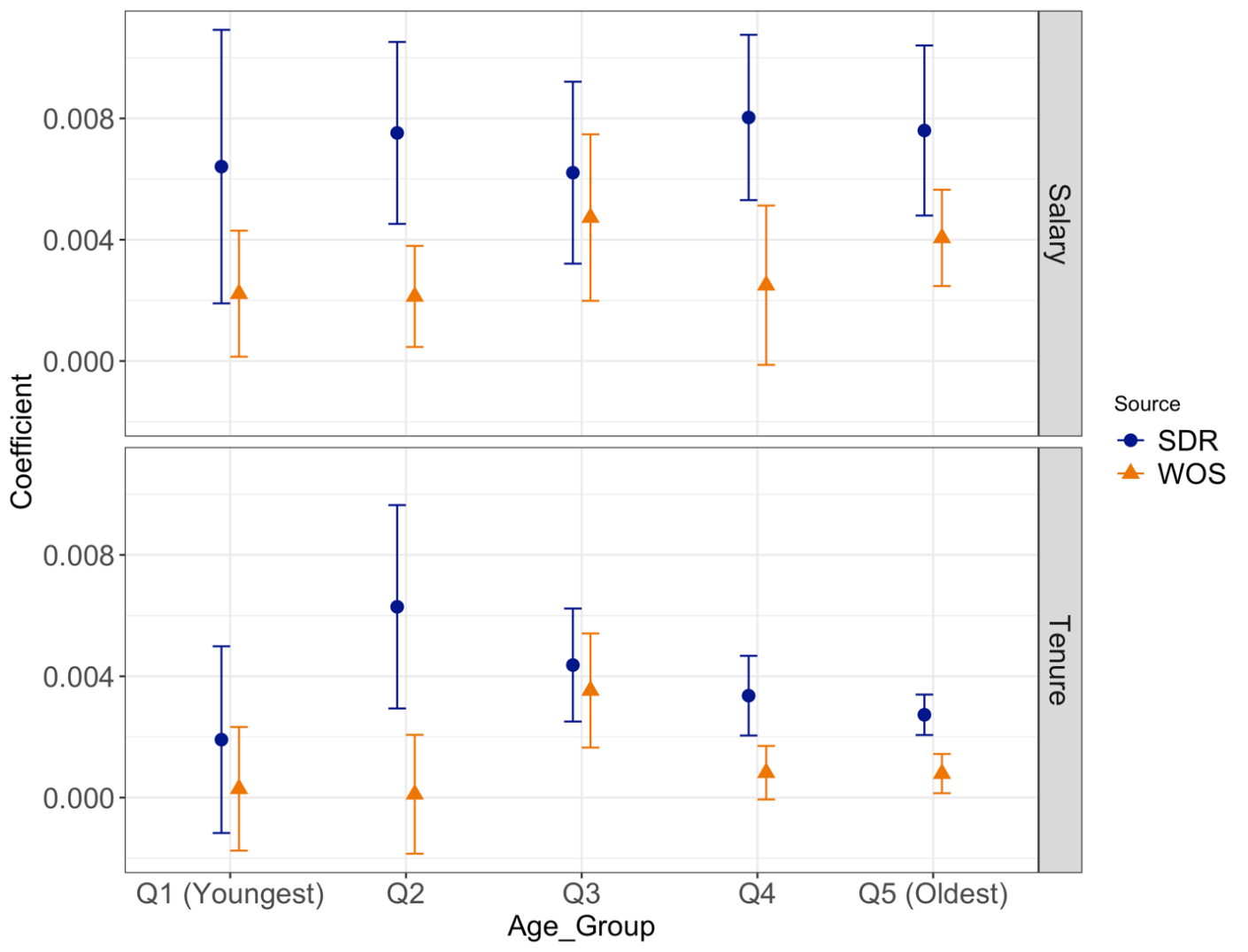


Fig A7: Relationship between Outcomes and SDR and WoS Publication Counts by Quintiles of Academic Age (2003—2008)

Notes: This figure plots the coefficients and 95% confidence intervals of the coefficients from regressions of ln(Salary) (upper panel) and an indicator for tenure status (lower panel) on publication counts from the SDR and WoS using 2008 SDR data. Each panel reports estimates from 5 separate regressions stratified by academic age. From the left to right, the bins are [1,4], [5,9], [10,16], [17,25], [26,45].


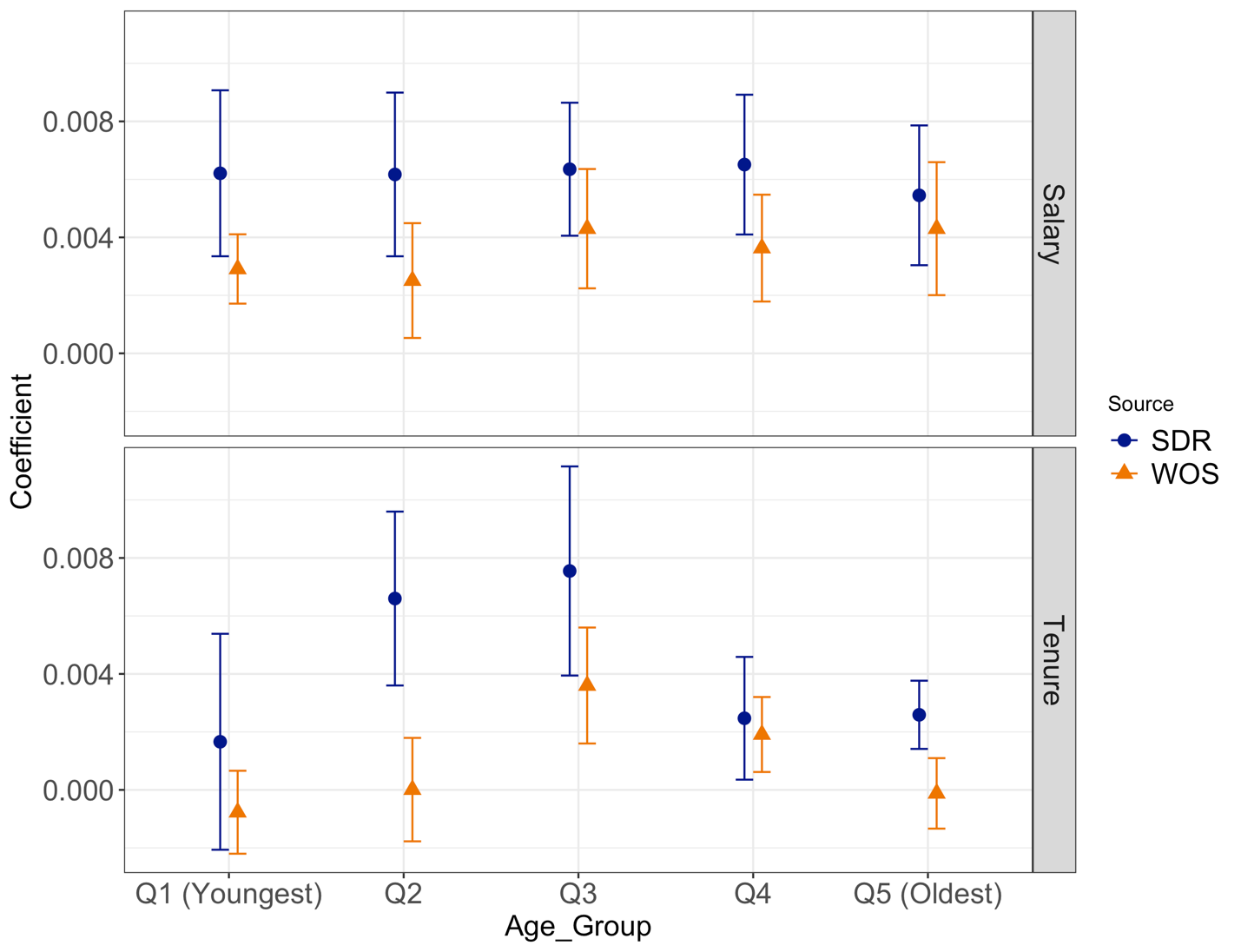


Fig A8: Relationship between Outcomes and SDR and WoS Publication Counts by Quintiles of Academic Age (1990—1995)

Notes: This figure plots the coefficients and 95% confidence intervals of the coefficients from regressions of ln(Salary) (upper panel) and an indicator for tenure status (lower panel) on publication counts from the SDR and WoS using 1995 SDR data. Each panel reports estimates from 5 separate regressions stratified by academic age. From the left to right, the bins are [1,4], [5,9], [10,16], [17,25], [26,45].


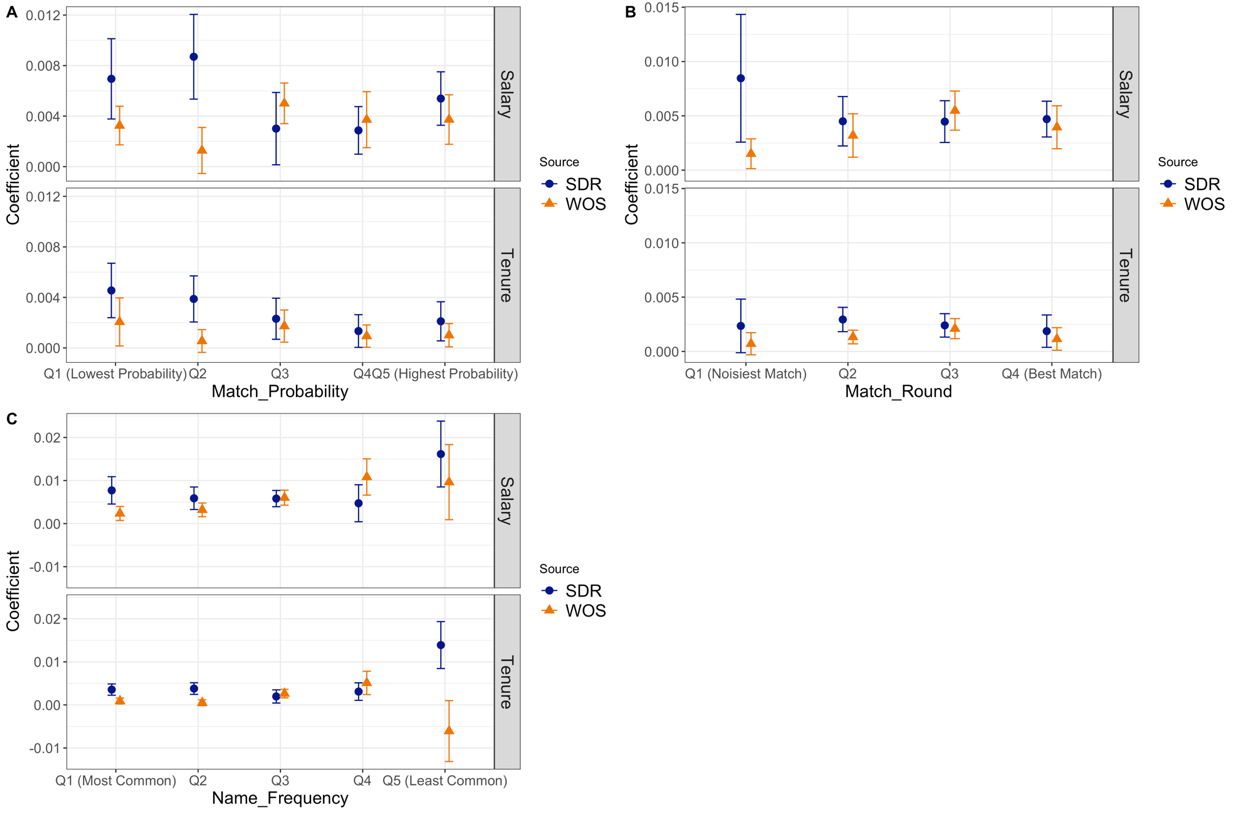


Fig A9: Relationship between Outcomes and SDR and WoS Publication Counts by Match Quality Measures (2003—2008)

Notes: Each sub-figure plots the coefficients and 95% confidence intervals of the coefficients from regressions of ln(Salary) (upper panel) and an indicator for tenure status (lower panel) on publication counts from the SDR and WoS using 2008 SDR data. Each panel reports estimates from 4 or 5 separate regressions stratified by measures of match quality. In sub-figure A, estimates are stratified by the mean match probability among all of a respondent’s publications. From the left to right, the bins are [0.5000, 0.6901], [0.6902, 0.7568], [0.7568, 0.8128], [0.8128, 0.8693], [0.8693, 1]. In sub-figure B, the estimates are stratified by the mean match round of an author’s publications with lower match rounds representing higher confidence. From the left to the right, the bins are [1.768, 2], [1.3076, 1.767], [1, 1.3076], [0, 1]. (We stratify the data by quartiles instead of quintiles because the first quartile has a large cluster at 1.) In sub-figure C, the estimates are stratified by name frequency (first initial and last name of the SDR respondent), with higher frequency names being more ambiguous. From the left to the right, the bins are [1423, 218422], [282, 1423], [83, 281], [25, 82], [1, 24].


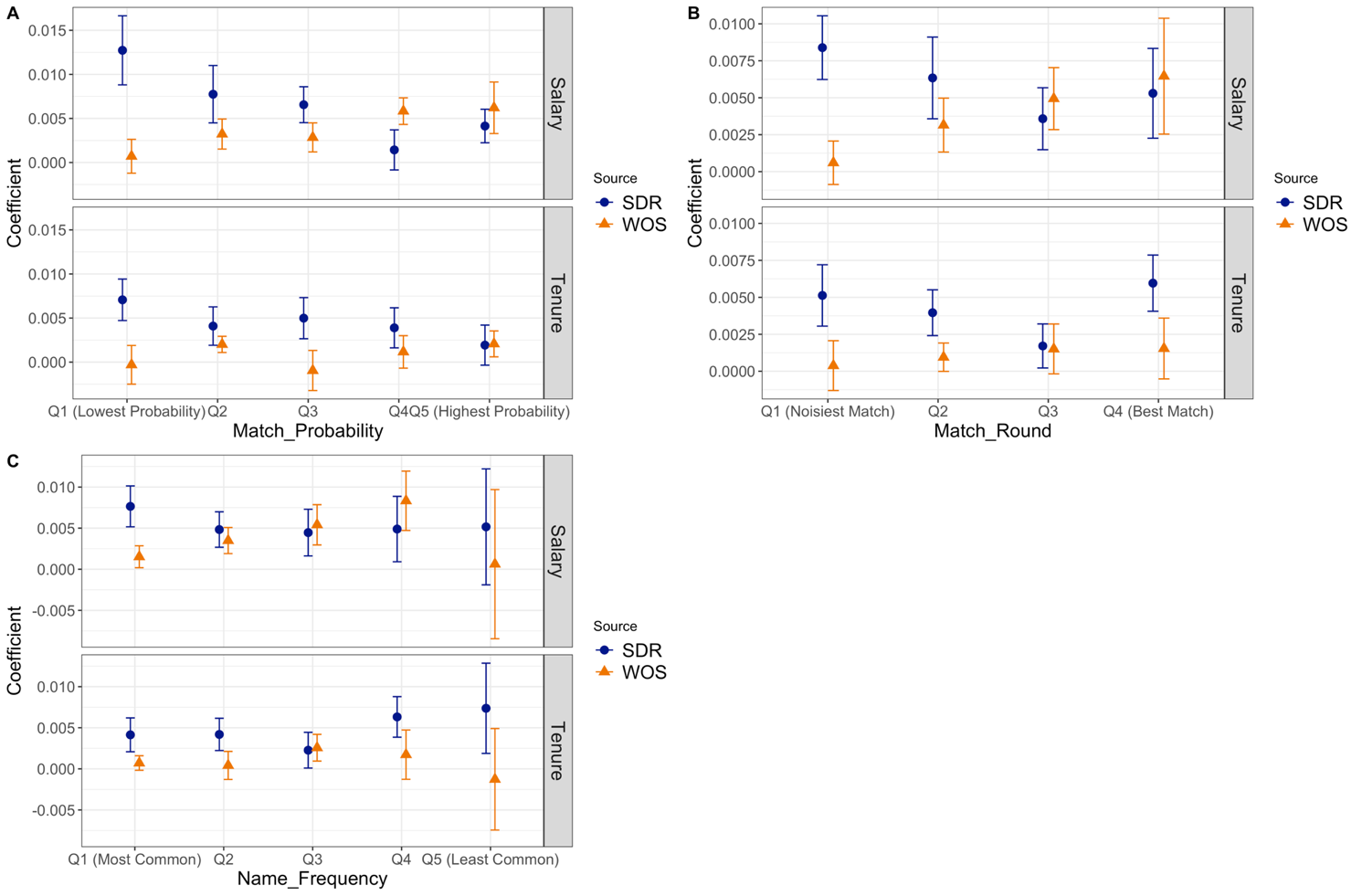


Fig A10: Relationship between Outcomes and SDR and WoS Publication Counts by Match Quality Measures (1990—1995)

Notes: Each sub-figure plots the coefficients and 95% confidence intervals of the coefficients from regressions of ln(Salary) (upper panel) and an indicator for tenure status (lower panel) on publication counts from the SDR and WoS using 1995 SDR data. Each panel reports estimates from 4 or 5 separate regressions stratified by measures of match quality. In sub-figure A, estimates are stratified by the mean match probability among all of a respondent’s publications. From the left to right, the bins are [0.5000, 0.6901], [0.6902, 0.7568], [0.7568, 0.8128], [0.8128, 0.8693], [0.8693, 1]. In sub-figure B, the estimates are stratified by the mean match round of an author’s publications with lower match rounds representing higher confidence. From the left to the right, the bins are [1.768, 2], [1.3076, 1.767], [1, 1.3076], [0, 1]. (We stratify the data by quartiles instead of quintiles because the first quartile has a large cluster at 1.) In sub-figure C, the estimates are stratified by name frequency (first initial and last name of the SDR respondent), with higher frequency names being more ambiguous. From the left to the right, the bins are [1423, 218422], [282, 1423], [83, 281], [25, 82], [1, 24].

Table A1. Summary Statistics, 2008 and 1995 Sample

|  | **2008 Sample** | | | | **1995 Sample** | | | |
| --- | --- | --- | --- | --- | --- | --- | --- | --- |
| **Variable** | **Obs** | **Weight** | **Mean** | **Std. Dev.** | **Obs** | **Weight** | **Mean** | **Std. Dev.** |
| SDR Pub | 10,626 | 257276.0 | 8.8 | 12.9 | 10,656 | 157465.7 | 8.9 | 10.5 |
| WoS Pub | 10,626 | 257276.0 | 12.2 | 20.9 | 10,656 | 157465.7 | 12.0 | 15.9 |
| Salary | 10,626 | 257276.0 | 89680.4 | 54891.2 | 10,656 | 157465.7 | 58471.6 | 29804.1 |
| Academic Age | 10,624 | 257214.4 | 15.8 | 11.5 | 10,404 | 154207.7 | 14.3 | 9.4 |
| Tenure Status | 10,626 | 257276.0 | 0.5419 | 0.4982 | 10,656 | 157465.7 | 0.5519 | 0.4973 |
| **Male** |  |  |  |  |  |  |  |  |
| SDR Pub | 6,543 | 170344.0 | 9.9 | 14.2 | 7,885 | 123261.8 | 9.6 | 11.1 |
| WoS Pub | 6,543 | 170344.0 | 14.0 | 23.0 | 7,885 | 123261.8 | 13.0 | 16.8 |
| **Female** |  |  |  |  |  |  |  |  |
| SDR Pub | 4,083 | 86932.0 | 6.7 | 9.5 | 2,771 | 34203.9 | 6.3 | 7.5 |
| WoS Pub | 4,083 | 86932.0 | 8.6 | 15.2 | 2,771 | 34203.9 | 8.1 | 10.8 |
| **Race = Asian** |  |  |  |  |  |  |  |  |
| SDR Pub | 796 | 8973.8 | 8.5 | 12.7 | 996 | 11721.4 | 10.9 | 12.0 |
| WoS Pub | 796 | 8973.8 | 9.8 | 17.0 | 996 | 11721.4 | 13.4 | 20.3 |
| **Race = Black** |  |  |  |  |  |  |  |  |
| SDR Pub | 690 | 8484.4 | 5.0 | 7.7 | 409 | 3119.6 | 5.8 | 7.0 |
| WoS Pub | 690 | 8484.4 | 6.3 | 12.1 | 409 | 3119.6 | 6.9 | 11.0 |
| **Race = Hispanic** |  |  |  |  |  |  |  |  |
| SDR Pub | 1,639 | 38373.8 | 9.4 | 12.3 | 477 | 3889.9 | 7.6 | 8.8 |
| WoS Pub | 1,639 | 38373.8 | 13.6 | 20.3 | 477 | 3889.9 | 9.0 | 12.4 |
| **Race = Others** |  |  |  |  |  |  |  |  |
| SDR Pub | 7,268 | 198002.9 | 8.9 | 13.1 | 74 | 585.2 | 6.5 | 5.8 |
| WoS Pub | 7,268 | 198002.9 | 12.2 | 21.1 | 74 | 585.2 | 8.8 | 11.9 |
| **Race = White** |  |  |  |  |  |  |  |  |
| SDR Pub | 233 | 3441.2 | 10.8 | 15.9 | 8,700 | 138149.6 | 8.9 | 10.4 |
| WoS Pub | 233 | 3441.2 | 15.5 | 33.6 | 8,700 | 138149.6 | 12.0 | 15.6 |
| **Faculty rank = “Other Faculty and Postdoc”** |  |  |  |  |  |  |  |  |
| SDR Pub | 2,370 | 55404.1 | 5.2 | 7.6 | 2,325 | 31686.8 | 6.1 | 7.6 |
| WoS Pub | 2,370 | 55404.1 | 7.4 | 11.3 | 2,325 | 31686.8 | 9.5 | 13.5 |
| **Faculty rank = “Assistant Professor”** |  |  |  |  |  |  |  |  |
| SDR Pub | 2,256 | 49496.2 | 7.0 | 8.0 | 2,353 | 30899.0 | 7.1 | 6.9 |
| WoS Pub | 2,256 | 49496.2 | 9.6 | 14.1 | 2,353 | 30899.0 | 9.4 | 10.9 |
| **Faculty rank = “Associate Professor”** |  |  |  |  |  |  |  |  |
| SDR Pub | 2,214 | 53177.7 | 8.4 | 10.5 | 2,452 | 35574.4 | 9.1 | 9.5 |
| WoS Pub | 2,214 | 53177.7 | 11.4 | 16.5 | 2,452 | 35574.4 | 11.7 | 14.5 |
| **Faculty rank = “Professor”** |  |  |  |  |  |  |  |  |
| SDR Pub | 3,259 | 86366.4 | 13.1 | 17.7 | 3,310 | 55583.8 | 11.6 | 13.3 |
| WoS Pub | 3,259 | 86366.4 | 18.1 | 29.3 | 3,310 | 55583.8 | 15.2 | 19.6 |

Notes: This table shows summary statistics of the key variables, respondent’s SDR and WoS publication counts, from the 2008 SDR (publication between 2003—2008) and 1995 SDR (publication between 1990—1995).

Table A2. Horse Race Comparisons of SDR and WoS Publication Counts as Determinants of Salaries, 2008 and 1995 Sample

|  | **2008 Sample** | | | | **1995 Sample** | | | |
| --- | --- | --- | --- | --- | --- | --- | --- | --- |
|  | (1) | (2) | (3) | (4) | (5) | (6) | (7) | (8) |
| SDR Pub | 0.008*** |  |  |  | 0.007*** |  |  |  |
|  | (0.001) |  |  |  | (0.001) |  |  |  |
| WoS Pub | 0.003*** |  |  |  | 0.004*** |  |  |  |
|  | (0.000) |  |  |  | (0.000) |  |  |  |
| ln(SDR Pub+1) |  | 0.096*** | 0.094*** |  |  | 0.083*** | 0.086*** |  |
|  |  | (0.007) | (0.010) |  |  | (0.006) | (0.007) |  |
| ln(WoS Pub+1) |  | 0.076*** | 0.086*** |  |  | 0.062*** | 0.061*** |  |
|  |  | (0.006) | (0.008) |  |  | (0.006) | (0.006) |  |
| ln(SDR Pub) |  |  |  | 0.079*** |  |  |  | 0.072*** |
|  |  |  |  | (0.008) |  |  |  | (0.006) |
| ln(WoS Pub) |  |  |  | 0.070*** |  |  |  | 0.049*** |
|  |  |  |  | (0.007) |  |  |  | (0.005) |
| Zero SDR Pub |  |  | -0.004 | -0.052** |  |  | 0.015 | -0.029 |
|  |  |  | -0.026 | -0.022 |  |  | (0.023) | (0.021) |
| Zero WoS Pub |  |  | 0.043* | (0.004) |  |  | - | - |
|  |  |  | -0.022 | -0.02 |  |  | - | - |
| Female | -0.089*** | -0.074*** | -0.074*** | -0.074*** | -0.122*** | -0.112*** | -0.112*** | -0.112*** |
|  | (0.014) | (0.013) | (0.013) | (0.013) | (0.013) | (0.013) | (0.013) | (0.013) |
| Marital status | 0.005 | 0.005 | 0.005 | 0.005 | 0.0006 | -0.0006 | -0.001 | -0.001 |
|  | (0.005) | (0.004) | (0.005) | (0.005) | (0.014) | (0.014) | (0.014) | (0.014) |
| Asian | 0.03222 | 0.04085 | 0.042 | 0.043 | -0.024 | -0.021 | -0.021 | -0.021 |
|  | (0.036) | (0.034) | (0.034) | (0.034) | (0.016) | (0.017) | (0.017) | (0.017) |
| Black | 0.074 | 0.100** | 0.101** | 0.102** | 0.044** | 0.063*** | 0.063*** | 0.063*** |
|  | (0.046) | (0.046) | (0.046) | (0.046) | (0.019) | (0.019) | -0.019 | -0.019 |
| Hispanic | 0.073 | 0.064 | 0.065 | 0.066 | 0.006 | 0.013 | 0.013 | 0.013 |
|  | (0.044) | (0.043) | (0.043) | (0.043) | (0.018) | (0.017) | -0.017 | -0.018 |
| Others | 0.036 | 0.036 | 0.036 | 0.037 | -0.021 | -0.021 | -0.02 | -0.022 |
|  | (0.040) | (0.039) | (0.039) | (0.039) | (0.039) | (0.039) | (0.039) | (0.039) |
| Observations | 9984 | 9984 | 9984 | 9984 | 9947 | 9947 | 9947 | 9947 |
| R-squared | 0.288 | 0.313 | 0.314 | 0.312 | 0.331 | 0.337 | 0.337 | 0.336 |
| Adjusted R-squared | 0.278 | 0.304 | 0.304 | 0.302 | 0.322 | 0.329 | 0.329 | 0.327 |

Notes: The dependent variable in this table is the natural log of the self-reported salary in 2008 (column 1-4) and in 1995 (column 5-8). The OLS specification in the four columns of each sample correspond to the OLS specification in column (5), (6), and (7) and (8) of Table 2. *p<0.1, **p<0.05, ***p<0.01. All standard errors are clustered at the academic age level.

Table A3. Horse Race Comparisons of SDR and WoS Publication Counts as Determinants of Tenure Status, 2008 and 1995 Sample

|  | **2008 Sample** | | | | **1995 Sample** | | | |
| --- | --- | --- | --- | --- | --- | --- | --- | --- |
|  | (1) | (2) | (3) | (4) | (5) | (6) | （7） | (6) |
| SDR Pub | 0.004*** |  |  |  | 0.005*** |  |  |  |
|  | (0.001) |  |  |  | (0.001) |  |  |  |
| WoS Pub | 0.001*** |  |  |  | 0.0008** |  |  |  |
|  | (0.000) |  |  |  | (0.000) |  |  |  |
| ln(SDR Pub+1) |  | 0.053** | 0.045*** |  |  | 0.061*** | 0.060*** |  |
|  |  | (0.005) | (0.008) |  |  | (0.009) | (0.009) |  |
| ln(WoS Pub+1) |  | 0.024*** | 0.027*** |  |  | 0.013** | 0.013* |  |
|  |  | (0.005) | (0.008) |  |  | (0.006) | (0.007) |  |
| ln(SDR Pub) |  |  |  | 0.037*** |  |  |  | 0.049*** |
|  |  |  |  | (0.006) |  |  |  | (0.007) |
| ln(WoS Pub) |  |  |  | 0.022*** |  |  |  | 0.011* |
|  |  |  |  | (0.007) |  |  |  | (0.005) |
| Zero SDR Pub |  |  | -0.031* | -0.055*** |  |  | -0.004 | -0.036* |
|  |  |  | (0.017) | (0.014) |  |  | (0.022) | (0.021) |
| Zero WoS Pub |  |  | 0.006 | -0.01 |  |  | - | - |
|  |  |  | (0.019) | (0.016) |  |  | - | - |
| Female | -0.043*** | -0.036*** | -0.036*** | -0.037*** | -0.045*** | -0.040*** | -0.040*** | -0.040*** |
|  | (0.010) | (0.009) | (0.009) | (0.009) | (0.014) | (0.014) | (0.014) | (0.014) |
| Marital status | 0.007 | 0.007 | 0.007 | 0.007 | -0.020** | -0.020** | -0.020** | -0.020** |
|  | (0.006) | (0.006) | (0.005) | (0.006) | (0.010) | (0.010) | (0.010) | (0.010) |
| Asian | 0.069** | 0.074*** | 0.074*** | 0.075*** | 0.005 | 0.005 | 0.005 | 0.005 |
|  | (0.027) | (0.026) | (0.026) | (0.026) | (0.012) | (0.011) | (0.011) | (0.011) |
| Black | 0.090*** | 0.103*** | 0.103*** | 0.104*** | 0.047** | 0.056*** | 0.056*** | 0.055*** |
|  | (0.027) | (0.027) | (0.027) | (0.027) | (0.018) | (0.018) | (0.018) | (0.018) |
| Hispanic | 0.044* | 0.042* | 0.042* | 0.043* | 0.028* | 0.031* | 0.031* | 0.031* |
|  | (0.024) | (0.024) | (0.024) | (0.024) | (0.017) | (0.017) | (0.017) | (0.017) |
| Others | 0.059** | 0.060** | 0.061** | 0.061** | -0.013 | -0.014 | -0.014 | -0.014 |
|  | (0.023) | (0.023) | (0.023) | (0.023) | (0.046) | (0.045) | (0.045) | (0.045) |
| Observations | 9502 | 9502 | 9502 | 9502 | 9759 | 9759 | 9759 | 9759 |
| R-squared | 0.483 | 0.490 | 0.491 | 0.490 | 0.501 | 0.505 | 0.505 | 0.504 |
| Adjusted R-squared | 0.475 | 0.483 | 0.483 | 0.482 | 0.495 | 0.499 | 0.498 | 0.498 |

Notes: The dependent variable in this table is an indicator of tenure status in 2008 (column 1-4) and in 1995 (column 5-8). The OLS specification in the four columns of each sample correspond to the OLS specification in column (5), (6), and (7) and (8) of Table 3. *p<0.1, **p<0.05, ***p<0.01. All standard errors are clustered at the academic age level.

Table A4: Instrumental Variable Analysis, 2008 and 1995 Sample

|  | **2008 Sample** | | | | **1995 Sample** | | | |
| --- | --- | --- | --- | --- | --- | --- | --- | --- |
|  | (1) | (2) | (3) | (4) | (5) | (6) | (7) | (8) |
| **Second Stage** | **Salary** | **Salary** | **Tenure** | **Tenure** | **Salary** | **Salary** | **Tenure** | **Tenure** |
|  |  |  |  |  |  |  |  |  |
| SDR Pub | 0.016*** |  | 0.006*** |  | 0.015*** |  | 0.007*** |  |
|  | (0.002) |  | (0.001) |  | (0.001) |  | (0.001) |  |
| WoS Pub |  | 0.011*** |  | 0.005*** |  | 0.011*** |  | 0.006*** |
|  |  | (0.001) |  | (0.001) |  | (0.001) |  | (0.001) |
|  |  |  |  |  |  |  |  |  |
| N | 10101 | 10101 | 9778 | 9778 | 9947 | 9947 | 9759 | 9759 |
| R-sq | 0.157 | 0.146 | 0.476 | 0.463 | 0.316 | 0.31 | 0.5 | 0.489 |
| adj. R-sq | 0.146 | 0.134 | 0.468 | 0.455 | 0.307 | 0.302 | 0.494 | 0.482 |
|  |  |  |  |  |  |  |  |  |
| **First Stage** | **SDR Pub** | **WoS Pub** | **SDR Pub** | **WoS Pub** | **SDR Pub** | **WoS Pub** | **SDR Pub** | **WoS Pub** |
| SDR Pub |  | 1.048 |  | 1.049 |  | 0.953 |  | 0.955 |
|  |  | (0.047) |  | (0.047) |  | (0.055) |  | (0.055) |
| WoS Pub | 0.417 |  | 0.418 |  | 0.421 |  | 0.421 |  |
|  | (0.019) |  | (0.020) |  | (0.018) |  | (0.018) |  |
| **F-stat of the First Stage** | 807.32 | 1762.56 | 863.08 | 1715.97 | 40.49 | 29.86 | 40.06 | 29.87 |

Notes: This table shows results from an instrument variables analysis. The upper panel reports second stage estimates and the lower panel reports first stage estimates. Each column is a separate model. In columns (1), (2), (5) and (6), the outcome variable is ln(Salary). In columns (3), (4), (7) and (8), the outcome variable is tenure status. The models in columns (1), (3), (5), and (7) use WoS publication counts to instrument for SDR publication counts. The models in columns (2), (4), (6), and (8) use SDR publication counts to instrument for WoS publication counts. Columns (1)—(4) use the 2008 SDR sample and columns (5)—(8) use the 1995 SDR sample.
